# Synthesis and Mechanochemical Activity of Peptide-Based Cu(I) Bis(*N*-heterocyclic carbene) Complexes

**DOI:** 10.3390/biomimetics4010024

**Published:** 2019-03-14

**Authors:** Sebastian Funtan, Philipp Michael, Wolfgang H. Binder

**Affiliations:** Macromolecular Chemistry, Institute of Chemistry, Faculty of Natural Science II, Martin Luther University Halle-Wittenberg, Von-Danckelmann-Platz 4, 06120 Halle (Saale), Germany; sebastian.funtan@chemie.uni-halle.de (S.F.); philipp.michael@chemie.uni-halle.de (P.M.)

**Keywords:** mechanochemistry, mechanocatalysts, “click” chemistry, peptide coupling, copper(I)

## Abstract

With the class of shock-absorbing proteins, nature created some of the most robust materials combining both mechanical strength and elasticity. Their excellent ability to dissipate energy to prevent surrounding cells from damage is an interesting property that regularly is exploited for applications in biomimetic materials. Similar to biomaterials, where mechanical stimuli are transmitted into a (bio)chemical response, mechanophoric catalysts transform mechanical energy into a chemical reaction. Force transmission is realized commonly by polymeric handles directing the applied force to the mechanophoric bond, which in turn leads to stress-induced activation of the catalyst. Therefore, shock-absorbing proteins able to take up and store mechanical energy elastically for subsequent force transduction to the labile bond seem to be perfect candidates to fulfill this task. Here, we report on the synthesis of two different latent mechanophoric copper(I) bis(*N*-heterocyclic carbene) complexes bearing either two carboxyl groups or two amino groups which allow conjugation reactions with either the N- or the C-terminus of amino acids or peptides. The chosen catalysts can be activated, for instance, by applying external mechanical force via ultrasound, removing one *N*-heterocyclic carbene (NHC) ligand. Post-modification of the mechanophoric catalysts via peptide coupling (Gly, Val) and first reactions showed that the mechanoresponsive behavior was still present after the coupling. Subsequent polycondensation of both catalysts lead to a polyamide including the Cu(I) moiety. Mechanochemical activation by ultrasound showed conversions in the copper(I)-catalyzed alkyne-azide “click” reaction (CuAAC) up to 9.9% proving the potential application for the time and spatial controlled CuAAC.

## 1. Introduction

Even though the rupture of macromolecules using strong shear forces has been already described by Staudinger in 1930 [1] and Eyring in 1940 [2], mechanochemistry is a comparably new field of research investigating the impact of mechanical forces to defined chemical changes in macromolecules [3,4,5]. Introducing selectively cleavable bonds into polymers enables chain scission in a chemically productive use via mechanochemistry even allowing reaction pathways to be altered [6]. Mechanophores are used for a widespread range of mechanochemical transformations such as color changes [7], changes in fluorescence [8,9,10], activation of latent catalysts [9,11], biased reactivity [6], release of small molecules [12], generation of protons [13], stabilization of radicals [14], remodeling of polymers [15,16], and also for molecular synthesis [17]. Exploiting such transformations, mostly in the fields of self-healing, stress sensing and catalysis can lead to a redirection of mechanochemistry from being “destructive to (being) productive” [18].

**Figure 1:**
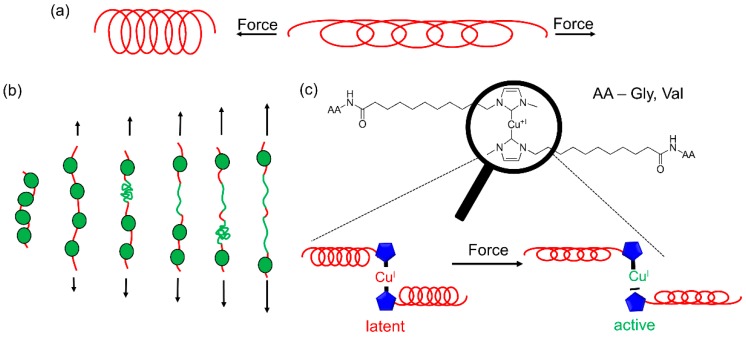
Energy dissipation mechanisms in proteins. (**a**) “Molecular spring” behavior and (**b**) stepwise unfolding of single protein domains under stress. (**c**) Top: Molecular structure of the low molecular weight catalysts bearing an amino acid (glycine or l-valine). Bottom: Exploitation of the “molecular spring” behavior for an efficient force transmission to facilitate the cleavage of the Cu–carbene bond.

Mechanochemistry is also omnipresent in nature [19] where it is involved in numerous vital processes such as cell growth [20], activation of ion channels [21,22], blood clotting [23], the sense of touch [24] and spatial orientation [25]. Also biomedical applications are an ongoing field of research to take advantage of these processes: for example, it is known that the in vitro differentiation of mesenchymal stem cells into neurons, myoblasts and osteoblasts is strongly influenced by the elasticity of the chosen matrix which may have implications for tissue engineering [26,27,28]. Mechanochemistry also plays a crucial role in several defensive mechanisms of humans and animals. Talin, titin, elastin and resilin are proteins belonging to the class of shock-absorbing proteins that dissipate mechanical force to protect cells from damage [29,30,31,32,33,34]. Even if the exact mechanism of those shock-absorbing proteins has not been fully understood yet, an elastic unfolding and refolding of those proteins under the influence of mechanical stress is assumed, with them acting as “molecular springs” (see Figure 1a), which was already proven for titin domains resulting in a saw-tooth pattern by atomic force microscopy (AFM) (Figure 1b) [29].

Apart from all similarities, there is a striking difference between the force necessary to trigger mechanochemical processes in nature and in synthetic molecules. Activating artificial (polymeric) systems by ultrasound or compression requires usually molecular forces in the range of 400 to 6000 pN [35,36,37,38], while in nature much weaker forces in the range of a few pN, far away from covalent bond dissociation energies, are sufficient for a mechanochemical activation, which underscores the term “soft-mechanochemistry” for those systems [39,40,41,42]. In addition to their reversibility, those systems offer a higher energy efficiency being advantageous for biological applications. Contrary to classical mechanochemistry, where usually specific bonds are ruptured, in soft-mechanochemistry the whole protein is involved in the mechanical activation, often leading to a conformational change within the protein, which ultimately will convert the external force into a biochemical signal [43]. It was found that the conformational change is not limited to single proteins but can also occur for supramolecular protein structures [44,45]. Some effort has been put into the synthesis of such artificial cryptic site exhibition systems that only allow ligand–receptor interaction upon stretching since they are shielded by polymer chains in the unstretched conformation [46,47,48,49,50], usually influencing the enzymatic catalytic activity [39].

**Scheme 1:**
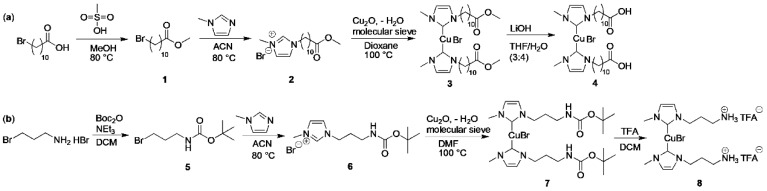
Pathway for the synthesis of both mechanophoric catalysts **4** and **8** bearing either (**a**) two carboxyl or (**b**) two amino groups.

In the present paper, we demonstrate the incorporation of mechanophoric latent Cu(I) bis(*N*-heterocyclic carbene) catalysts [9] to single amino acids and peptides (Figure 1c). A set of various mechanophoric catalysts bearing different end groups is generated, which opens the opportunity for peptide coupling to exploit their excellent spring-like elastic properties. Beside the well-known key factors of an efficient force transmission via long, force-transmitting polymer handles and the correct positioning of the labile copper–carbene bond in the center of the molecule [51,52], the application of peptides offers the additional advantage of mechanochemical not distortive amide bonds preventing their mechanocatalytical activation. However, it is reported that ultrasound can affect the secondary structure of peptides through conformational changes [53]. Even though a lot of effort has been put into mechanochemistry in the last decade, surprisingly few attempts have been made to realize mechanophoric catalysts bearing additional, non-mechanoresponsive functionalities [4,5]. Here, we developed a synthetic route for imidazolium-based bifunctional Cu(I)-catalysts bearing either two carboxyl groups ([Cu(C_10_COOH-NHC)_2_]Br) or two amino groups ([Cu(C_3_NH_2_-NHC)_2_]Br) (for structures, see Scheme 1) which allow classical peptide coupling with the N-terminus as well as the C-terminus of a peptide. Ultrasonication was used as a tool for mechanochemical activation by an external force. Furthermore, polymeric structures were investigated by coupling the two bifunctional ([Cu(C_10_COOH-NHC)_2_]Br) and ([Cu(C_3_NH_2_-NHC)_2_]Br) complexes.

## 2. Materials and Methods

### 2.1. Materials

All chemicals were purchased from Sigma-Aldrich (Munich, Germany), Tokyo Chemical Industry Co., Ltd. (Eschborn, Germany), Carl Roth GmbH & Co. KG (Karlsruhe, Germany), Grüssing GmbH (Karlsruhe, Germany), Carbolution Chemicals GmbH (St. Ingbert, Germany), Fluka (Bucharest, Romania), Alfa Aesar (Karlsruhe, Germany), Chemotrade GmbH (Düsseldorf, Germany), or VWR BDH Prolabo (Darmstadt, Germany); and were used without further purification. Glycine methyl ester hydrochloride (**9**) was synthesized according to standard procedures [54], while l-valine methyl ester hydrochloride (**10**) was synthesized as described elsewhere [55]. The synthesis of benzyl azide was done analogous to literature [56]. 1,4-Dioxane, acetonitrile (ACN), dichloromethane (DCM), dimethylformamide (DMF) and methanol were dried with calcium hydride, while tetrahydrofuran (THF) was dried with sodium/benzophenone. All solvents were freshly distilled and degassed by bubbling with nitrogen for at least 20 min prior to use. For further details see Supplemental Materials.

### 2.2. Methods

^1^H nuclear magnetic resonance (NMR) and ^13^C NMR spectra were recorded either on a Varian Gemini 2000 (400 MHz; Agilent, Waldbronn, Germany) or on a Varian Unity Inova 500 MHz spectrometer (Agilent) using MestReNova software (version 6.0.2-5475, Mestrelab Research, Santiago de Compostela, Spain) for the evaluation of the results. The spectra were measured at 27 °C using deuterated chloroform (CDCl_3_), deuterated dimethyl sulfoxide (DMSO-d_6_) or deuterated tetrahydrofuran (THF-d_8_). All chemical shifts (δ) were given in parts per million (ppm) and were referred to the solvent residual signal (CDCl_3_: 7.26 ppm (^1^H NMR), 77.0 ppm (^13^C NMR); DMSO-d_6_: 2.50 ppm (^1^H NMR), 39.5 ppm (^13^C NMR); THF-d_8_: 1.72 ppm (^1^H NMR), 67.2 ppm (^13^C NMR)). Subscript letters of protons (*H*_y_) and carbons (*C*_y_) indicate the corresponding atoms within the structures shown on the ^1^H NMR and ^13^C NMR spectra.

Gel permeation chromatography (GPC) measurements were performed on a Viscotek GPCmax VE 2001 (Malvern Panalytical Ltd., Crowthorne, UK) using a H_HR_-H Guard-17369 (Malvern Panalytical Ltd.) and a GMH_HR_-N-18055 column (Malvern Panalytical Ltd.) with DMF containing 10 mM LiTf_2_N as eluent at 60 °C and via detection of the refractive index with a Viscotek VE 3580 RI detector (Malvern Panalytical Ltd.) at 35 °C. The external calibration was done using polystyrene standards (MP = 1000–115,000 g/mol). The concentration of all samples was 5 mg/mL and the flow rate was 1 mL/min.

Thin-layer chromatography (TLC) was performed using Merck silica gel 60 plates (Merck, Darmstadt, Germany). Spots on the TLC plate were visualized using an oxidizing agent blue stain or ultraviolet (UV) light (254 or 366 nm). The blue staining solution was prepared as follows: (NH_4_)_6_Mo_7_O_24_∙4H_2_O (1 g) and Ce(SO_4_)_2_∙4H_2_O (1 g) were dissolved in a mixture of distilled water (90.0 mL) and concentrated sulfuric acid (6.0 mL).

Column chromatography was carried out using silica gel high-purity grade, 60 Å pore size, 230–400 mesh particle size (Merck).

### 2.3. Synthesis

*Synthesis of 11-bromoundecanoic acid methyl ester* (**1**): 11-Bromoundecanoic acid (11.15 g, 42.01 mmol) was dissolved in MeOH (120.0 mL) and subsequently catalytic amounts of methanesulfonic acid were added. The mixture was refluxed for 48 h at 80 °C. Afterward, the solvent was removed, the crude product was redissolved in Et_2_O (80.0 mL) and washed with saturated NaHCO_3_ (40.0 mL), water (40.0 mL) and brine (40.0 mL). The organic phase was dried with MgSO_4_, filtered and the solvent was removed to yield the product as a yellow liquid. Yield: 11.1 g, 39.8 mmol, 95%.

^1^H NMR (400 MHz, CDCl_3_, 27 °C): δ (ppm) = 3.66 (s, 3H), 3.40 (t, 2H), 2.30 (t, 2H), 1.84 (m, 2H), 1.61 (m, 2H), 1.41 (m, 2H), 1.28 (m, 10H); ^13^C NMR (100 MHz, CDCl_3_, 27 °C): δ (ppm) = 174.3, 51.4, 34.1, 34.0, 32.8, 29.3, 29.3, 29.2, 29.1, 28.7, 28.1, 24.9.

*Synthesis of 3-(11-methoxy-11-oxoundecyl)-1-methyl-1*H*-imidazolium bromide* (**2**): 11-Bromoundecanoic acid methyl ester (**1**) (7.9 g, 28.27 mmol) and 1-methylimidazole (1.93 g, 1.88 mL, 23.56 mmol) were dissolved in ACN (16.0 mL). The reaction mixture was stirred for 48 h at 80 °C. Afterward, the solvent was removed and the crude product was stored in the fridge until complete crystallization. The solid product was washed with Et_2_O (5 × 20.0 mL), the solvent was removed and the pure product was dried by rotary evaporation. Yield: 8.22 g, 22.8 mmol, 97%.

^1^H NMR (400 MHz, CDCl_3_, 27 °C): δ (ppm) = 10.51 (s, 1H), 7.45 (s, 1H), 7.33 (s, 1H), 4.30 (t, 2H), 4.12 (s, 3H), 3.64 (s, 3H), 2.28 (t, 2H), 1.88 (m, 2H), 1.58 (m, 2H), 1.25 (m, 12H); ^13^C NMR (100 MHz, CDCl_3_, 27 °C): δ (ppm) = 174.2, 137.8, 123.2, 121.6, 51.4, 50.1, 36.7, 34.0, 30.2, 29.1, 29.1, 29.0, 29.0, 28.8, 26.1, 24.8; mass spectrometry (MS) (electrospray ionization (ESI)), *m/z* calculated for [C_16_H_29_N_2_O_2_]^+^ = 281.224; found 281.222; infrared (IR): ν_max_ (cm^−1^): 3418 (w), 3063 (w), 2923 (m), 2853 (m), 1730 (s), 1562 (m), 1469 (m), 1418 (m), 1361 (m), 1335 (m), 1305 (w), 1272 (w), 1240 (m), 1207 (m), 1162 (s), 1110 (w), 1035 (w), 1014 (w), 986 (w), 969 (w), 881 (m), 832 (s), 786 (w), 744 (m), 722 (m), 654 (w), 617 (s).

*Synthesis of bis(1-methylimidazol-3-(11-methoxy-11-oxoundecyl) ylid-2-ene)copper(I) bromide complex [Cu(C_10_COOMe-NHC)_2_]Br* (**3**): 3-(11-Methoxy-11-oxoundecyl)-1-methyl-1*H*-imidazolium bromide (**2**) (1.0 g, 2.76 mmol), Cu_2_O (1.97 g, 13.61 mmol) and activated molecular sieve (3 Å) were suspended in 1,4-dioxane (10.0 mL) and were refluxed for 68 h. The solution was filtered and the solvent was removed. Further purification was done via column chromatography on silica changing the eluent polarity gradually from pure CHCl_3_ (*R_f_* = 0.1), over CHCl_3_/MeOH 40:1 (*v*/*v*) (*R_f_* = 0.33) to CHCl_3_/MeOH 20:1 (*v*/*v*) (*R_f_* = 0.55). After removal of the solvent the product was obtained as a colorless, crystalline solid. Yield: 397.0 mg, 0.56 mmol, 41%.

^1^H NMR (400 MHz, CDCl_3_, 27 °C): δ (ppm) = 6.15 (dd, 2H), 3.65 (s, 3H), 3.58 (t, 2H), 3.25 (s, 3H), 2.29 (t, 2H), 1.62 (m, 4H), 1.26 (m, 12H); ^13^C NMR (100 MHz, CDCl_3_, 27 °C): δ (ppm) = 174.3, 153.2, 111.0, 109.9, 51.4, 43.6, 34.1, 30.3, 29.5, 29.4, 29.3, 29.2, 29.1, 26.6, 24.9; IR: ν_max_ (cm^−1^): 2919 (m), 2850 (m), 1729 (s), 1664 (s), 1489 (m), 1463 (m), 1448 (m), 1424 (m), 1390 (w), 1379 (w), 1365 (w), 1271 (m), 1244 (w), 1205 (m), 1171 (w), 1113 (m), 982 (w), 767 (w), 673 (s), 652 (m), 556 (w).

*Deprotection of [Cu(C_10_COOMe-NHC)_2_]Br* (**4**) [57]: Lithium hydroxide monohydrate (24.4 mg, 0.58 mmol) was dissolved in water (4.0 mL) and was added to the methyl ester-protected catalyst **3** (100.0 mg, 0.142 mmol) while keeping the temperature at 0 °C. After stirring for 5 min, THF (3.0 mL) was added until all reagents were dissolved. The reaction was allowed to warm to room temperature and was followed via TLC (CHCl_3_/MeOH (20:1), *R_f_* = 0.24) until no starting material was present anymore (CHCl_3_/MeOH (20:1), *R_f_* = 0.38). After 4 h the pH was adjusted to 4 by adding 1 M HCl. After extraction with CHCl_3_ (1 × 25.0 mL, 4 × 12.5 mL), the combined organic phases were dried over Na_2_SO_4_, filtered and the solvent was removed. The pure product was obtained as a slightly yellow solid. Yield: 92.03 mg, 0.137 mmol, 96%.

^1^H NMR (400 MHz, CDCl_3_, 27 °C): δ (ppm) = 6.17 (dd, 2H), 3.59 (m, 2H), 3.26 (s, 3H), 2.31 (t, 2H), 1.62 (m, 4H), 1.28 (m, 12H); ^13^C NMR (100 MHz, CDCl_3_, 27 °C): δ (ppm) = 177.8, 153.2, 111.3, 110.2, 43.7, 34.1, 30.5, 29.4, 29.1, 29.0, 28.9, 28.9, 28.8, 26.4, 24.7; IR: ν_max_ (cm^−1^): 2918 (s), 2851 (s), 1718 (s), 1627 (s), 1486 (m), 1469 (m), 1446 (w), 1385 (w), 1304 (w), 1267 (w), 1258 (w), 1240 (w), 1203 (m), 1173 (s), 961 (w), 890 (w), 815 (s), 774 (w), 645 (s).

*Synthesis of* N*-(*tert*-butoxycarbonyl)-3-bromopropylamine* (**5**) [58]: 3-Bromopropan-1-amine hydrobromide (6.55 g, 30.0 mmol) was suspended in DCM (12.0 mL) and the mixture was cooled down to 0 °C followed by the addition of triethylamine (3.49 g, 4.78 mL, 34.5 mmol). Afterward, a solution of di-*tert*-butyl dicarbonate (5.04 g, 23.0 mmol) in DCM (12.5 mL) was added to the reaction mixture and stirred for 24 h at room temperature. Subsequently, the solution was washed with HCl (1M, 4 × 20.0 mL) and the remaining organic phase was washed with brine (20.0 mL) and dried over Na_2_SO_4_. After filtration and removal of the solvent the pure product was obtained as a slightly yellow oil that crystallized in the freezer. Yield: 5.30 g, 22.2 mmol, 97%.

^1^H NMR (400 MHz, CDCl_3_, 27 °C): δ (ppm) = 4.65 (s, 1H), 3.43 (t, 2H), 3.27 (dd, 2H), 2.04 (p, 2H), 1.44 (s, 9H); ^13^C NMR (100 MHz, CDCl_3_, 27 °C): δ (ppm) = 155.9, 79.4, 39.0, 32.7, 30.8, 28.4; IR: ν_max_ (cm^−1^): 2965 (s), 1669 (s), 1487 (s), 1448 (m), 1374 (w), 1295 (s), 1240 (w), 1114 (s), 1074 (s), 994 (w), 947 (w), 908 (w), 817 (w), 764 (s), 682 (m), 555 (m).

*Synthesis of 3-[3-[[(1,1-dimethylethoxy)carbonyl]amino]propyl]-1-methyl-1*H*-imidazolium bromide* (**6**): *N*-(*tert*-butoxycarbonyl)-3-bromopropylamine (**5**) (5.30 g, 22.2 mmol) and 1-methylimidazole (2.19 g, 2.13 mL, 26.7 mmol) were dissolved in ACN (12.2 mL) and the reaction mixture was stirred for 40 h at 80 °C. Afterward, the solvent was removed and the crude product was redissolved in water (20.0 mL) and washed with CHCl_3_ (10 × 15.0 mL) and EtOAc (10 × 15.0 mL). Subsequently, the water was removed, the product was redissolved in CHCl_3_ and dried over Na_2_SO_4_. Filtration and removal of the solvent yielded the pure product as a yellow, highly viscous liquid. Yield: 5.67 g, 17.7 mmol, 80%.

^1^H NMR (400 MHz, CDCl_3_, 27 °C): δ (ppm) = 10.30 (s, 1H), 7.63 (s, 1H), 7.38 (s, 1H), 5.66 (s, 1H), 4.41 (t, 2H), 4.07 (s, 3H), 3.18 (dd, 2H), 2.15 (m, 2H), 1.41 (s, 9H); ^13^C NMR (100 MHz, CDCl_3_, 27 °C): δ (ppm) = 156.6, 138.0, 123.1, 122.7, 79.4, 47.5, 36.7, 30.6, 28.4; MS (ESI), *m/z* calculated for [C_12_H_22_N_3_O_2_]^+^ = 240.172; found 240.171; IR: ν_max_ (cm^−1^): 3265 (m), 2976 (m), 1690 (s), 1572 (w), 1514 (m), 1454 (w), 1391 (w), 1364 (m), 1270 (m), 1250 (m), 1163 (s), 1005 (w), 853 (w), 752 (w), 651 (m), 620 (m).

*Synthesis of bis(1-methylimidazol-3-[3-(((1,1-dimethylethoxy)carbonyl)amino)propyl] ylid-2-ene) copper(I) bromide complex [Cu(C_3_NHBoc-NHC)_2_]Br* (**7**): 3-[3-[[(1,1-Dimethylethoxy)carbonyl]amino]propyl]-1-methyl-1*H*-imidazolium bromide (**6**) (0.83 g, 2.58 mmol), Cu_2_O (1.85 g, 12.90 mmol) and activated molecular sieve (3 Å) were suspended in DMF (5.0 mL) and were refluxed for 70 h. The solution was filtered afterward and the solvent was removed. Further purification was done via column chromatography on silica changing the solvent polarity gradually from pure CHCl_3_ (*R_f_* = 0.09) to CHCl_3_/MeOH 60:1 (*v*/*v*) (*R_f_* = 0.17). After removal of the solvent the product was obtained as a yellow, sticky liquid that slowly crystallized. Yield: 159 mg, 0.32 mmol, 25%.

^1^H NMR (400 MHz, CDCl_3_, 27 °C): δ (ppm) = 6.18 (dd, 2H), 5.45 (s, 1H), 3.67 (t, 2H), 3.25 (s, 3H), 3.08 (dd, 2H). 1.75 (m, 2H), 1.42 (s, 9H); ^13^C NMR (100 MHz, CDCl_3_, 27 °C): δ (ppm) = 156.1, 153.6, 111.6, 110.1, 78.9, 40.5, 36.8, 30.4, 29.9, 28.4; IR: ν_max_ (cm^−1^): 3289 (w), 2971 (w), 1694 (w), 1661 (s), 1517 (m), 1475 (w), 1443 (w), 1412 (w), 1390 (w), 1364 (w), 1348 (w), 1279 (m), 1249 (m), 1222 (w), 1163 (m), 1137 (m), 1049 (w), 974 (m), 945 (m), 853 (w), 819 (w), 783 (w), 748 (w), 679 (s), 608 (s), 586 (w).

*Deprotection of [Cu(C_3_NHBoc-NHC)_2_]Br* (**8**) [57]: The Boc-protected complex **7** (50.0 mg, 0.10 mmol) was dissolved in DCM (500.0 µL). The mixture was cooled down to 0 °C with an ice bath before trifluoroacetic acid (TFA) (289.3 mg, 195.5 µL, 2.54 mmol) was added. The mixture was left to stir at room temperature and completion of the reaction was followed via TLC (CHCl_3_/MeOH (20:1), *R_f_* = 0.04) until no starting material was visible anymore (CHCl_3_/MeOH (20:1), *R_f_* = 0.56). After complete consumption of the starting material, the solvent and the TFA were removed in vacuo to yield the product as a yellow, sticky liquid that slowly crystallized. Yield: 51.1 mg, 0.08 mmol, 98%.

^1^H NMR (400 MHz, DMSO-d_6_, 27 °C): δ (ppm) = 7.80 (s, 3H), 6.49 (dd, 2H), 3.57 (t, 2H), 3.10 (s, 3H), 2.75 (m, 2H). 1.82 (m, 2H); ^13^C NMR (100 MHz, DMSO-d_6_, 27 °C): δ (ppm) = 153.2, 112.3, 110.7, 40.3, 36.7, 30.3, 27.7; IR: ν_max_ (cm^−1^): 2958 (m), 1719 (s), 1672 (s), 1597 (m), 1481 (s), 1443 (w), 1411 (m), 1377 (w), 1304 (w), 1251 (w), 1169 (s), 1111 (s), 1019 (m), 995 (m), 850 (m), 830 (m), 810 (m), 795 (m), 784 (m), 762 (w), 750 (w), 719 (s), 649 (s), 632 (m), 595 (w), 552 (w).

*Synthesis of glycine methyl ester modified [Cu(C_10_COOH-NHC)_2_]Br* (**11**): The deprotected complex **4** (18.10 mg, 26.9 µmol) was dissolved in DCM (500.0 µL). The mixture was cooled down to 0 °C in an ice bath, and molecular sieve (3 Å) and *N*,*N*′-dicyclohexylcarbodiimide (DCC) (12.22 mg, 59.2 µmol) were added. The mixture was stirred for 10 min before pentafluorophenol (10.89 mg, 59.2 µmol) was added. After stirring for another 15 min the glycine methyl ester hydrochloride (**9**) (7.50 mg, 53.8 µmol) was dissolved in DCM (500.0 µL) and *N*,*N*-diisopropylethylamine (DIPEA) (15.30 mg, 20.06 µL, 0.1184 mmol) and was also added to the reaction. After stirring for 2 h at 0 °C, the ice bath was removed and the mixture was further stirred for 20 h until completion of the reaction (CHCl_3_/MeOH (20:1), *R_f_* = 0.29). The formed precipitate was removed by filtration and the product was concentrated in vacuo. Purification by column chromatography (CHCl_3_, *R_f_* = 0.1) yielded the pure product as a white, crystalline solid. Yield: 11.9 mg, 15.0 µmol, 54%.

^1^H NMR (400 MHz, CDCl_3_, 27 °C): δ (ppm) = 6.15 (dd, 2H), 6.06 (s, 1H), 4.03 (d, 2H), 3.75 (s, 3H), 3.57 (m, 2H), 3.23 (s, 3H), 2.23 (m, 2H), 1.63 (m, 4H), 1.27 (m, 12H); ^13^C NMR (100 MHz, CDCl_3_, 27 °C): δ (ppm) = 173.3, 170.6, 153.2, 111.1, 109.9, 52.3, 43.6, 41.1, 36.3, 30.3, 29.5, 29.3, 29.2, 29.2, 29.1, 29.1, 26.5, 25.5; IR: ν_max_ (cm^−1^): 3324 (m), 3131 (w), 2919 (s), 2849 (m), 1744 (s), 1684 (s), 1637 (s), 1541 (s), 1471 (s), 1458 (w), 1437 (m), 1412 (m), 1370 (m), 1353 (m), 1305 (w), 1253 (m), 1240 (m), 1208 (s), 1184 (s), 1115 (w), 1095 (w), 1052 (w), 1042 (w), 1010 (w), 974 (w), 898 (w), 806 (w), 772 (w), 753 (w), 718 (w), 665 (s), 634 (w), 590 (w), 574 (w).

*Synthesis of l-valine methyl ester modified [Cu(C_10_COOH-NHC)_2_]Br* (**12**): The deprotected complex **4** (50.0 mg, 74.3 µmol) was dissolved in DCM (1.0 mL). The mixture was cooled down to 0 °C in an ice bath, and molecular sieve (3 Å) and 1-ethyl-3-(3-dimethylaminopropyl)carbodiimide hydrochloride (EDC·HCl) (31.34 mg, 163.5 µmol) were added. The mixture was stirred for 10 min before pentafluorophenol (30.09 mg, 163.5 µmol) was dissolved in DCM (200.0 µL) and added to the solution. After stirring for another 15 min, the l-valine methyl ester hydrochloride (**10**) (24.91 mg, 148.6 µmol) was dissolved in DCM (500.0 µL) and was added together with DIPEA (63.38 mg, 83.40 µL, 490.0 µmol) to the reaction. After stirring for 2 h at 0 °C, the ice bath was removed and the mixture was stirred for 70 h until completion of the reaction (TLC control; CHCl_3_/MeOH (20:1 (*v*/*v*)), *R_f_* = 0.43). Water (10.0 mL) was added to the reaction and the organic phase was washed with HCl (1M, 4 × 5.0 mL), saturated NaHCO_3_ solution (3 × 5.0 mL) and brine (5.0 mL). The organic phase was dried with Na_2_SO_4_, filtered and the solvent was removed to obtain the product as a slightly yellow, viscous liquid. Yield: 28.8 mg, 33.0 µmol, 45%.

^1^H NMR (400 MHz, CDCl_3_, 27 °C): δ (ppm) = 6.13 (dd, 2H), 5.96 (d, 1H), 4.55 (dd, 2H), 3.71 (s, 3H), 3.54 (m, 2H), 3.22 (s, 3H), 2.21 (m, 2H), 2.13 (dtd, 1H), 1.61 (m, 4H), 1.27 (m, 12H), 0.89 (dd, 6H); ^13^C NMR (100 MHz, CDCl_3_, 27 °C): δ (ppm) = 173.0, 172.7, 153.3, 111.1, 110.0, 56.8, 52.1, 43.7, 36.7, 31.3, 30.3, 29.5, 29.3, 29.3, 29.2, 29.1, 29.1, 26.5, 25.6, 18.9, 17.8; IR: ν_max_ (cm^−1^): 3284 (w), 2926 (m), 2854 (m), 1743 (m), 1655 (s), 1533 (m), 1468 (m), 1411 (w), 1372 (w), 1328 (m), 1202 (m), 1153 (m), 1002 (w), 658 (m), 553 (w).

*Coupling of [Cu(C_10_COOH-NHC)_2_]Br and [Cu(C_3_NH_2_-NHC)_2_]Br to the polymeric catalyst* (**13**): The COOH-functionalized complex **4** (31.09 mg, 46.0 µmol) and DIPEA (13.08 mg, 16.63 µL, 100.0 µmol) were dissolved in DMF (200.0 µL) followed by the addition of molecular sieve (3 Å) and DCC (56.95 mg, 276.0 µmol) in the countercurrent of nitrogen. The mixture was stirred for 5 min before pentafluorophenol (50.80 mg, 276.0 µmol) was added. Subsequently, the NH_2_-functionalized complex **8** (29.9 mg, 46.0 µmol) and DIPEA (13.08 mg, 16.63 µL, 100.0 µmol) were dissolved in DMF (150.0 µL) and added to the mixture, which was stirred afterwards for 75 h at 30 °C. After the reaction was finished, CHCl_3_ (2.0 mL) was added and the solution was filtered. The crude product was purified via column chromatography on silica changing the solvent polarity gradually from pure CHCl_3_ (*R_f_* = 0.0), over CHCl_3_/MeOH 40:1 (*v*/*v*) (*R_f_* = 0.20) to CHCl_3_/MeOH 20:1 (*v*/*v*) (*R_f_* = 0.42).

^1^H NMR (400 MHz, CDCl_3_, 27 °C): δ (ppm) = 7.02 (t, N*H*), 6.20 (s, 2H), 6.16 (dd, 2H), 3.67 (m, 2H), 3.56 (t, 2H), 3.26 (s, 3H), 3.23 (s, 3H), 3.17 (dd, 2H), 2.18 (t, 2H), 1.76 (dt, 2H), 1.61 (m, 4H), 1.26 (m, 12H); ^13^C NMR (100 MHz, CDCl_3_, 27 °C): δ (ppm) = 173.6, 153.6, 153.1, 111.9, 111.1, 110.2, 110.0, 43.6, 40.5, 36.9, 35.2, 30.5, 30.3, 29.5, 29.3, 29.3, 29.2, 29.2, 29.2, 29.1, 26.5, 25.7; IR: ν_max_ (cm^−1^): 3268 (w), 2925 (m), 2853 (w), 1655 (s), 1545 (m), 1472 (m), 1410 (w), 1377 (w), 1239 (m), 1200 (w), 1129 (w) 826 (w), 660 (s).

### 2.4. Mechanochemical Activation

Ultrasonication experiments were conducted by placing the latent mechanocatalyst (7.50 µmol) into a 10 mL reaction vessel with two additional side necks attached to a VCX 500 ultrasonic processor (Sonics & Materials, Inc., Newtown, CT, USA) equipped with a long full wave solid probe and an internally threaded stainless steel adapter. The vessel was evacuated and flushed with nitrogen at least three times to remove oxygen. Subsequently, benzyl azide (103.0 mg, 97.0 µL, 750.0 µmol) and phenylacetylene (77.0 mg, 82.0 µL, 750.0 µmol) were added as well as 10.0 mL of a THF-d_8_/MeOH mixture (30:1 (*v*/*v*)) which resulted in a catalyst concentration of 0.75 mM. To the mixture, successive cycles of pulsed ultrasound with a frequency of 20 kHz using 20% of the maximal amplitude with a sequence of 5 s pulse and 10 s break for 90 min were applied corresponding to an ultrasound power intensity of 4.66 W/cm^2^ and an energy input of 11 kJ. During this time the mixture was cooled in a water bath to prevent the temperature from rising above 25 °C. Each cycle was followed by a waiting time of 60 min. Samples were taken after the cycles 0, 3, 5, 10, 14 and 17 and the conversion of the “click” reaction was checked via ^1^H NMR spectroscopy by observing the increase in the triazole resonance at 8.11 ppm as well as the shift of the methylene group from 4.35 to 5.59 ppm.

Control experiments without ultrasound were carried out in two-necked flasks at room temperature as well as at 60 °C to prove the activation of the catalyst by ultrasound.

## 3. Results and Discussion

### 3.1. Synthesis of the Mechanophoric Catalysts

We started with the synthesis of Cu(I) bis(NHC) complexes, bearing appropriate functional groups to allow a subsequent extension by amino acids via peptide-coupling. Therefore two different Cu(I) bis(NHC) complexes were designed: one bearing two carboxylic acid moieties (**4**), the second bearing two terminal amino groups (**8**), both allowing peptide/protein attachment via the N- or C-terminus of amino acids/peptides. The carboxyl-functionalized complex ([Cu(C_10_COOH-NHC)_2_]Br) (**4**) was synthesized in a four-step synthesis (Scheme 1a). In a first step, 11-bromoundecanoic acid was protected via a methyl ester to prevent a complexation with the copper(I) in the following steps which would lower the yield of the desired complex significantly (5–10%, see Supplementary Materials). The obtained product **1** was subsequently quaternized using *N*-methylimidazole forming thus the NHC precursor **2** in quantitative yields which in turn is able to act as ligand for the desired Cu(I) complexes. For deprotonation and complexation, Cu_2_O was chosen as internal base and copper(I) source at once [59] due to the lability of the methyl ester group under basic conditions realizing thus the synthesis of the [Cu(C_10_COOMe-NHC)_2_]Br complex (**3**) instead of the commonly used method which involves strong bases such as potassium bis(trimethylsilyl)amide (KHMDS) or sodium *tert*-butoxide [9]. Finally, LiOH was used for deprotection yielding the carboxyl-functionalized catalyst [Cu(C_10_COOH-NHC)_2_]Br (**4**) quantitatively. 

**Figure 2:**
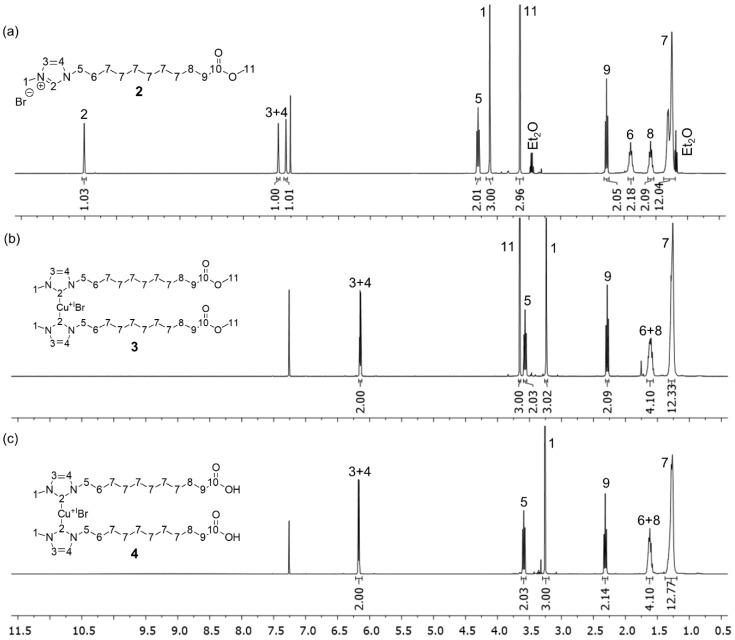
^1^H NMR spectra of (**a**) the NHC precursor **2**, (**b**) the methyl ester protected complex **3,** and (**c**) the deprotected complex **4** bearing two carboxyl groups.

The successful synthesis of complex **4** was proven comparing the ^1^H NMR spectrum of the imidazolium precursor **2** (Figure 2a) with the ^1^H NMR spectrum of the protected catalyst **3** (Figure 2b) and deprotected [Cu(C_10_COOH-NHC)_2_]Br catalyst (**4**) (Figure 2c). The absence of proton resonances *H*_2_ (–NC*H*N–) at 10.51 ppm from precursor **2** to complex **3** proved together with the significant shift of the protons *H*_3_ and *H*_4_ (–NC*H*=C*H*N–) from 7.45 ppm and 7.33 ppm to 6.15 ppm the successful deprotonation and formation of the Cu(I) bis(NHC) complex **3** and is thus in good compliance with literature [60]. Comparing these ^1^H NMR spectra with those of the deprotected catalyst **4** revealed the successful removal of the protection group due to the absence of protons *H*_11_ (–C(O)OC*H*_3_) at 3.64 ppm, while the signals *H*_3_ and *H*_4_ (–NC*H*=C*H*N–) at 6.15 ppm stay unaffected. Together with the absence of *H*_2_ (–NC*H*N–) at 10.51 ppm, it could be shown that no decomposition and re-protonation of the complex took place. 

For the synthesis of the amine-functionalized ([Cu(C_3_NH_2_-NHC)_2_]Br) complex (**8**) (Scheme 1b), 3-bromopropan-1-amine hydrobromide was protected in a first step with a Boc group to avoid side reactions with the primary amines during the subsequent complex formation and enabling an efficient purification process. The Boc-protected imidazolium precursor **6** was obtained after a quaternization with *N*-methylimidazole in a yield of 80% and was subsequently transformed to the [Cu(C_3_NHBoc-NHC)_2_]Br complex (**7**) using the same Cu_2_O method described above. The deprotection was achieved with trifluoroacetic acid yielding the amino-functionalized complex [Cu(C_3_NH_2_-NHC)_2_]Br (**8**) quantitatively. 

**Figure 3:**
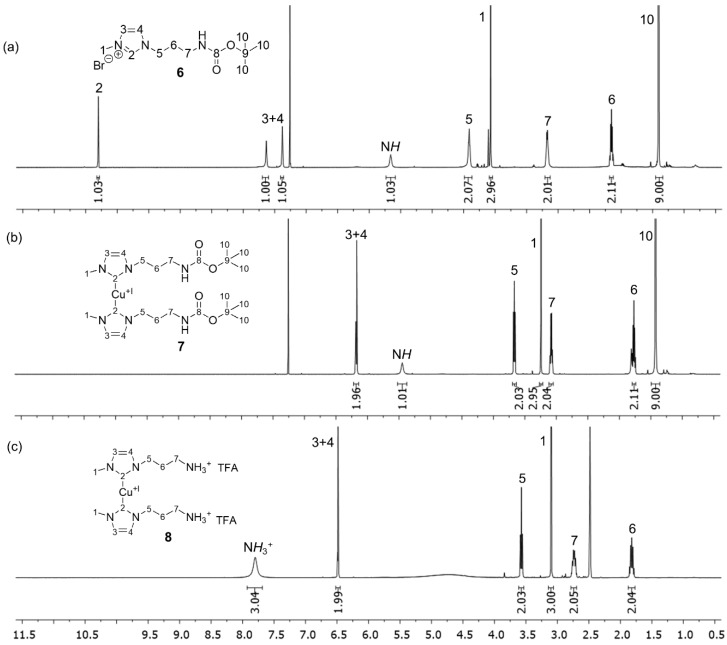
^1^H NMR spectra of (**a**) the NHC precursor **6**, (**b**) the Boc-protected complex **7,** and (**c**) the deprotected complex **8** bearing two amino groups.

Similar to the previously described biscarboxyl-telechelic catalyst **4**, the absence of the resonances of proton *H*_2_ (–NC*H*N–) at 10.30 ppm in the ^1^H NMR spectra (Figure 3a,b) verified the successful formation of the Cu–carbene bond. The shift of protons *H*_3_ and *H*_4_ (–NC*H*=C*H*N–) from 7.63 ppm and 7.38 ppm in case of the precursor **6** to 6.18 ppm for complex **7** proved the formation of the bis(NHC) complex. The ^1^H NMR spectrum of complex **8** (Figure 3c) revealed the successful removal of the Boc protection group which was no longer visible at 1.42 ppm. Moreover, the ^1^H NMR spectrum indicated that both amino groups of **8** are protonated after the deprotection with trifluoroacetic acid (resonance at 7.80 ppm corresponding to –NH_3_^+^TFA^−^).

### 3.2. Peptide Coupling Reactions

**Scheme 2:**
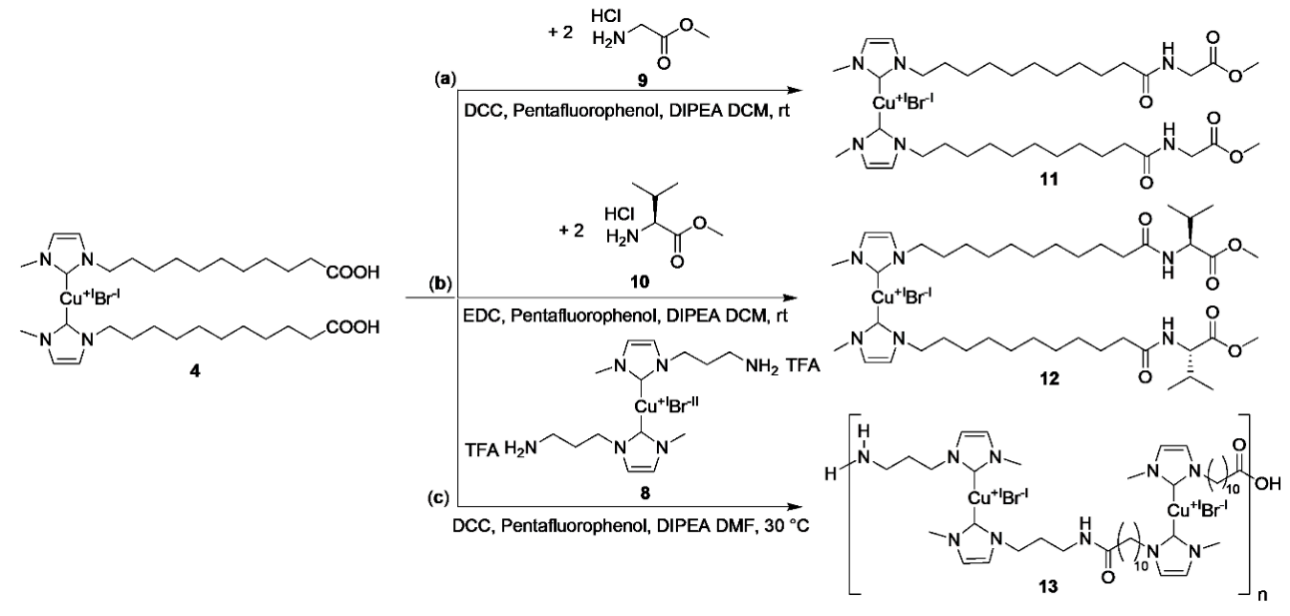
Performed peptide coupling reactions using the mechanophoric catalysts. (**a**) Coupling of methyl ester protected glycine (**9**) and (**b**) of methyl ester protected l-valine (**10**) to [Cu(C_10_COOH-NHC)_2_]Br (**4**). (**c**) The coupling of both the bifunctional carboxy (**4**) and bifunctional amino catalysts (**8**) generating a polymeric mechanocatalyst (**13**). rt: Room temperature.

Peptide coupling reactions were performed to probe the attachment of amino acids onto the Cu(I) bis(NHC)-complexes **4** and **8**. Firstly, it was crucial to understand if a proper attachment via conventional peptide coupling strategies could be effected. Secondly, it was important to investigate the effect of attached amino acids toward the catalytic activity of the mechanoresponsive copper(I) catalysts in CuAAC “click” reactions. Therefore, glycine methyl ester hydrochloride (**9**) and l-valine methyl ester hydrochloride (**10**) were coupled to catalyst **4** using common coupling agents [61,62] such as DCC or EDC·HCl and pentafluorophenol as shown in Scheme 2a,b. 

**Figure 4:**
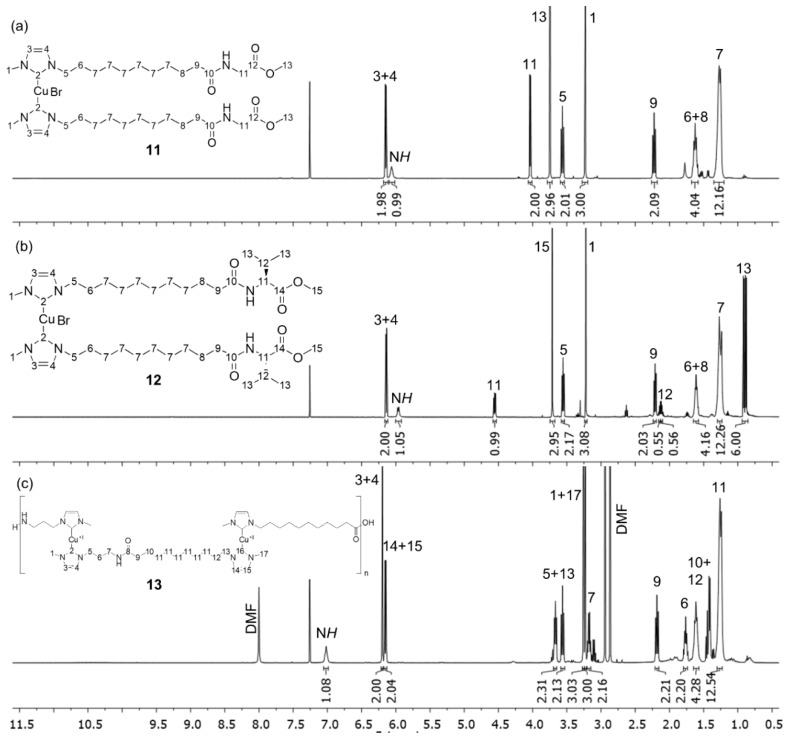
^1^H NMR spectra of (**a**) glycine-modified complex **11**, (**b**) l-valine-modified complex **12,** and (**c**) of the polymeric catalyst **13**.

Both, the glycine- (**11**) and the l-valine-modified mechanophores (**12**) could be obtained in yields of around 50%. The ^1^H NMR spectrum of catalyst **11** is shown in Figure 4a. The N*H* of the formed peptide bond was detected at 6.06 ppm, while protons *H*_11_ (–NHC*H*_2_–) and *H*_13_ (–OC*H*_3_) from the coupled glycine methyl ester were present at 4.03 and 3.75 ppm, respectively. The signals of protons *H*_3_ and *H*_4_ (–NC*H*=C*H*N–) of the Cu(I) NHC ring at 6.15 and 6.06 ppm verify that the complex was not destroyed during the coupling. Analogous the ^1^H NMR spectrum of the l-valine-modified complex (**12**) (Figure 4b) showed the successful formation of the peptide bond at 5.96 ppm (N*H*) as well as all signals belonging to the introduced l-valine.

**Figure 5:**
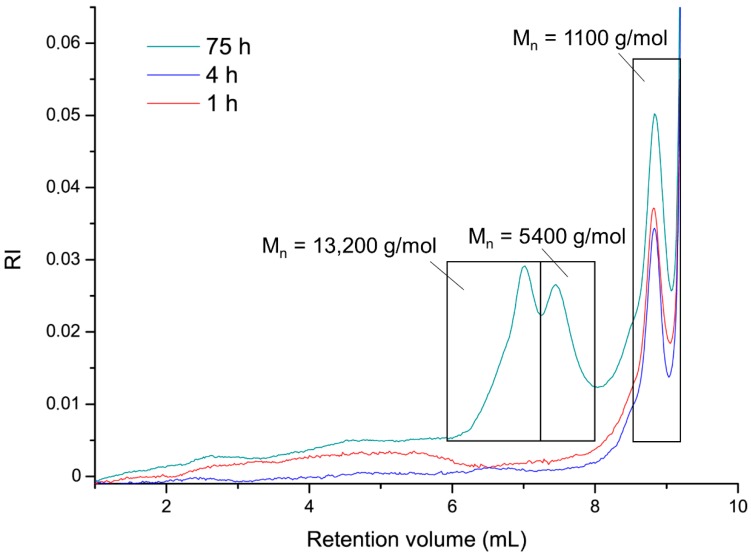
The peptide coupling between the two bifunctional catalysts **4** and **8** can be followed via GPC. It is expected that the reaction follows a step-growth mechanism due to its polycondensation character. This is proven by GPC where the formation of the dimer is observed early during the reaction. The formation of higher molecular weight species is only observed, if most of the monomer is consumed (75 h).

Extending the concept of peptide-linking to the Cu(I) bis(NHC) complexes and elongating the force transmitting polymeric scaffold with nondistortive peptide bonds, a coupling of dicarboxylic telechelic catalyst **4** with catalyst **8**, bearing two amino groups were performed (Scheme 2c). Due to the bifunctional character of both catalysts, an equimolar polycondensation under the formation of amide bonds took place, embedding the Cu(I) bis(NHC) complex within a polyamide chain to ensure remaining mechanoresponsive behavior [60]. The polycondensation was monitored via GPC (Figure 5) observing a dimeric structure formation (1100 g/mol) at the early stage of the reaction after 1 h, while higher condensates (5400 g/mol up to 13,200 g/mol) can be observed after ongoing reaction time (up to 75 h) due to the step-growth character of the polycondensation leading also to broader molecular weight distributions.

Figure 4c shows the purified ^1^H NMR spectrum of the coupled catalyst **13**. The signal of the N*H–* group at 7.02 ppm clearly showed the successful peptide coupling and, likewise, for the other catalysts the resonances at 6.20 and 6.16 ppm coming from the –NC*H*=C*H*N– protons of the catalysts (*H*_3_, *H*_4_, *H*_14_, and *H*_15_) proved the presence of the undestroyed Cu(I) bis(NHC) structure.

### 3.3. Investigation of Catalytic Activity of the Cu(I) bis(NHC) Complexes

**Figure 6:**
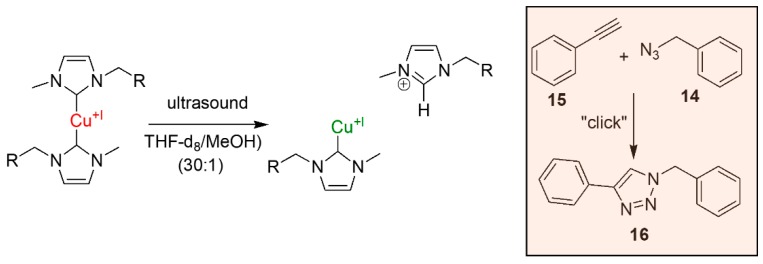
Activation of the latent catalyst is achieved by ultrasonication which cleaves one of the two shielding NHC ligands. This allows the alkyne to coordinate to the Cu(I) and the “click” reaction can take place. As a model reaction, the “click” reaction between benzyl azide (**14**) and phenylacetylene (**15**) was chosen.

Subsequently, the catalytic activity of the synthesized catalysts were investigated using a model CuAAC “click” reaction of benzyl azide (**14**) and phenylacetylene (**15**). Cu(I) bis(NHC) complexes are known for their mechanoresponsive behavior since they can be switched from their latent, inactive state to their active state after exposure to mechanical stress, for example in the form of ultrasound (in solution) or by compression (in bulk) [9,60,63]. In its latent state the Cu(I) center is surrounded by the two shielding NHC ligands which prevent the alkyne from coordination to the copper(I) center. When ultrasound is applied, one of the NHC ligands is cleaved from the Cu by rupturing the copper–carbene bond, in turn enabling the replacement by an alkyne under formation of the copper acetylide which is known to be the crucial step in the “click” reaction [64]. The principle of the catalyst activation is shown in Figure 6.

The activation of the latent catalyst to its active monocarbene form was observed by monitoring the subsequent CuAAC “click” reaction (see Figure 6) of **14** and **15** via ^1^H NMR spectroscopy. Observing the shifts from the origin methylene group of **14** from 4.35 to 5.59 ppm for the “click” product **16** as well as an increasing triazole resonance at 8.11 ppm enable the calculation of the “click” conversion in dependence of the applied sonication time.

**Figure 7:**
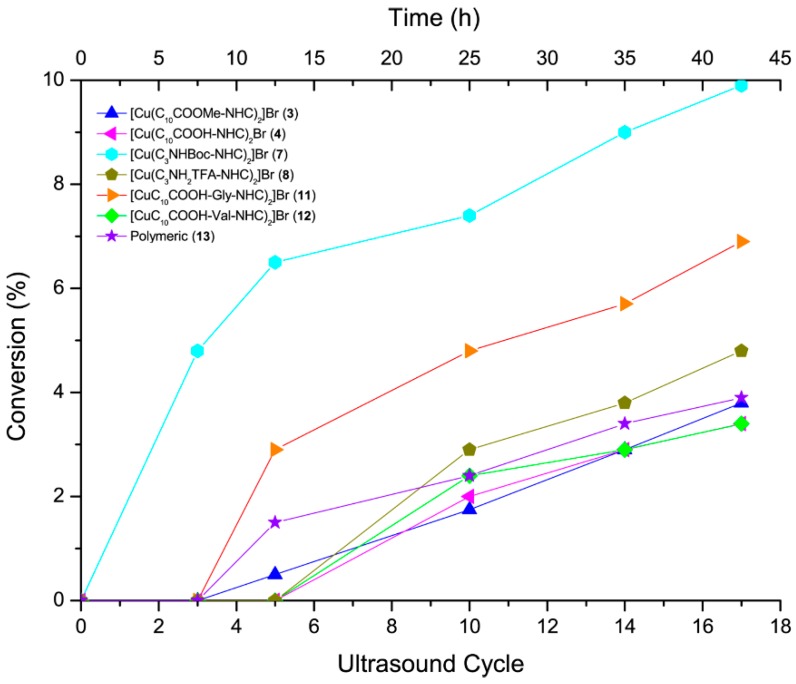
Percentage of conversion of phenylacetylene (**15**) and benzyl azide (**14**) to the “clicked” product **16** as calculated from ^1^H NMR spectroscopy with a standard deviation of ±1%. Samples were taken after the cycles 0, 3, 5, 10, 14, and 17.

In compliance with the literature [9], for all Cu(I) bis(NHC) catalysts an increase in the “click” conversion with increasing sonication time was observed (Table 1 and Figure 7). In case of the [Cu(C_10_COOMe-NHC)_2_]Br (**3**) and its deprotected pendant [Cu(C_10_COOH-NHC)_2_]Br (**4**) a similar catalytic activity could be determined after activation of the catalysts by ultrasound. Both catalysts showed a latency period up to the 5th sonication cycles. Thus, both catalysts revealed only a minimal conversion of 0.5% after the 5th cycle which increased afterward linearly up to a conversion of 3.5% after the 17th cycle. 

The Boc-protected amine-based catalyst [Cu(C_3_NHBoc-NHC)_2_]Br (**7**) showed a significantly higher activity than its deprotected pendant [Cu(C_3_NH_2_-NHC)_2_]Br (**8**). A conversion of 4.8% could already be detected after the 3rd cycle and increased linearly during the next cycles up to 9.9% after the 17th cycle. The deprotection of the catalyst to [Cu(C_3_NH_2_-NHC)_2_]Br (**8**) had a bigger influence on the mechanoresponsivity in comparison to [Cu(C_10_COOH-NHC)_2_]Br (**4**): an activation could only be detected after the 10th cycle (2.9% conversion) subsequently increasing linearly to a conversion of 4.8% after the 17th cycle which is only the half of the Boc-protected catalyst **7**. It can be hypothesized that the sterically demanding Boc groups caused a pre-stretching of the copper–carbene bond and facilitated its cleavage [60,65]. The higher conversion using the [Cu(C_3_NH_2_-NHC)_2_]Br catalyst (**8**) instead of the carboxyl-based catalyst (**4**) can be explained by the ionic end group structure (–NH_3_^+^TFA^−^; see Figure 2) of catalyst **8** which is able to facilitate the protonation of the cleaved free NHC and accelerates thus the copper(I) acetylide formation. The peptide coupling between [Cu(C_10_COOH-NHC)_2_]Br (**4**) and methyl ester-protected l-valine (**10**) leading to [Cu(C_10_COOH-Val-NHC)_2_]Br (**12**) does not affect the catalytic activity, which is still present in “click” conversions of 3.4% after the 17th cycle. Thus, a successful peptide coupling which involves several coupling agents as well as a base, could be performed, causing no deactivation of the Cu(I) bis(NHC) catalyst. Likewise, the methyl ester-protected glycine modified [Cu(C_10_COOH-Gly-NHC)_2_]Br catalyst (**11**) revealed catalytic activity which could be detected after the 5th cycle with a conversion of 2.9% increasing linearly to 6.9% after the 17th cycle.

To exclude undesired site reactions and proving the activation of the catalyst only by ultrasonication, test reactions were performed at room temperature using, apart from ultrasound, the same conditions for the “click” reaction. Therefore, mixtures containing the catalysts **3**, **4**, **7**, **8** and **12** were stirred at room temperature for 42.5 h observing in all cases no conversion. Additionally, reactions were conducted at 60 °C to exclude thermal influences on the catalyst activation revealing only very small conversions (1–2%) compared to the ultrasound-initiated “click” reactions in the range of uncatalyzed Huisgen 1,3-dipolar reactions [9,66]. 

The coupling of both bifunctional copper(I) complexes **4** and **8** yielded a polymeric catalyst **13** that still possessed mechanoresponsive behavior and only showed activation after application of ultrasound. Thus, the potential of peptide-based Cu(I) bis(NHC) complexes for a time and spatial controlled activation via mechanical force could be proven which opens the opportunity for a new class of mechanoresponsive molecules.

## 4. Conclusions

We successfully synthesized Cu(I) bis(NHC) mechanophoric catalysts bearing either two carboxyl groups (**4**) or two amino groups (**8**) and linked them via peptide bonds to the amino acids glycine (**11**) and l-valine (**12**). The chosen groups (–COOH and –NH_2_) are perfect candidates for subsequent modification reactions with peptides since they allow the coupling to both the C- and N-terminus. A direct peptide coupling between the carboxyl- (**4**) and amino-functionalized (**8**) Cu(I) bis(NHC) catalysts generated peptide-based mechanophores.

The mechanophoric behavior of the low molecular weight complexes was proven via ultrasonication both for the protected ([Cu(C_10_COOMe-NHC)_2_]Br (**3**), [Cu(C_3_NHBoc-NHC)_2_]Br (**7**)) and the unprotected catalysts ([Cu(C_10_COOH-NHC)_2_]Br (**4**), [Cu(C_3_NH_2_-NHC)_2_]Br (**8**)). While the deprotection of the [Cu(C_10_COOMe-NHC)_2_]Br (**3**) catalyst did not show significant impact on the mechanoresponsive behavior with a conversion around 3.5%, in both cases the deprotection of [Cu(C_3_NHBoc-NHC)_2_]Br (**7**) reduced conversion from 9.9% to 4.8% for [Cu(C_3_NH_2_-NHC)_2_]Br (**8**). The mechanoresponsive behavior was still present after coupling of single methyl ester protected amino acids to the [Cu(C_10_COOH-NHC)_2_]Br (**4**) complex. However, while the coupling of glycine led to a slightly higher conversion of 6.9% the coupling of l-valine did not increase the conversion which still was at 3.4%. The polymeric catalyst (**13**) still showed mechanoresponsive behavior after application of ultrasound opening thus the opportunity to couple not only single amino acids but also whole peptide chains to our low molecular weight catalysts. The coupling of peptides such as elastin and titin that are known for their elasticity and their “molecular spring” behavior will be exploited for an efficient force transmission along the backbone to facilitate catalyst activation. 

## Figures and Tables

**Table 1:** “Click” reactions between benzyl azide (**14**) and phenylacetylene (**15**) using ultrasound in the presence of mechanophoric catalysts. ^1^

Entry	Catalyst	Ultrasound ^2^	*t* (h)	*T* (°C)	Conversion ^3^ (%)
1	**3**	on	42.5	rt	3.8
2	**4**	on	42.5	rt	3.4
3	**4**	off	42.5	rt	0
4	**4**	off	42.5	60	1.9
5	**7**	on	42.5	rt	9.9
6	**8**	on	42.5	rt	4.8
7	**11**	on	42.5	rt	6.9
8	**12**	on	42.5	rt	3.4
9	**13**	on	42.5	rt	3.9

^1^ For all reactions 1.0 eq. of azide **14**, 1.0 eq. of alkyne **15**, and 0.01 eq. of the Cu(I) catalyst (0.75 mM) were used in THF-d_8_/MeOH (30:1 (*v*/*v*)). ^2^ 17 Cycles were performed where one cycle consisted of 90 min pulsing at 20 kHz with 20% amplitude of 125 µm with a pulse sequence of 5 s/pulse, 10 s/break followed by 60 min without pulsing. ^3^ The “click” reaction was followed by the increasing triazole resonance originating from product **16** in the ^1^H NMR at δ = 8.11 ppm as well as the shift from the methylene group of the benzyl azide (**14**) from δ = 4.35 ppm to δ = 5.59 ppm with a standard deviation of ±1%. rt: Room temperature.

## Supplementary Materials

The following are available online at https://www.mdpi.com/2313-7673/4/1/24/s1, Figure S1: ^1^H NMR of 11-bromoundecanoic acid methyl ester (**1**), Figure S2: ^13^C NMR of 11-bromoundecanoic acid methyl ester (**1**), Figure S3: ^1^H-NMR of 3-(11-methoxy-11-oxoundecyl)-1-methyl-1H-imidazolium bromide **2**; Figure S4: ^13^C-NMR of 3-(11-methoxy-11-oxoundecyl)-1-methyl-1*H*-imidazolium bromide (**2**), Figure S5: ^1^H NMR of [Cu(C_10_COOMe-NHC)_2_]Br (**3**), Figure S6: ^13^C NMR of [Cu(C_10_COOMe-NHC)_2_]Br (**3**), Figure S7: ^1^H NMR of [Cu(C_10_COOH-NHC)_2_]Br (**4**), Figure S8: ^13^C NMR of [Cu(C_10_COOH-NHC)_2_]Br (**4**), Figure S9: ^1^H NMR of *N*-*tert*-butoxycarbonyl)-3-bromopropylamine (**5**), Figure S10: ^13^C NMR of *N*-*tert*-butoxycarbonyl)-3-bromopropylamine (**5**), Figure S11: ^1^H NMR of 3-[3-[[(1,1-dimethylethoxy)carbonyl]amino]propyl]-1-methyl-1*H*-imidazolium bromide (**6**), Figure S12: ^13^C NMR of 3-[3-[[(1,1-dimethylethoxy)carbonyl]amino]propyl]-1-methyl-1*H*-imidazolium bromide (**6**), Figure S13: ^1^H NMR: of [Cu(C_3_NHBoc-NHC)_2_]Br (**7**), Figure S14: ^13^C NMR of [Cu(C_3_NHBoc-NHC)_2_]Br (**7**), Figure S15: ^1^H NMR spectrum [Cu(C_3_NH_2_-NHC)_2_]Br (**8**), Figure S16: ^13^C NMR spectrum [Cu(C_3_NH_2_-NHC)_2_]Br (**8**), Figure S17: ^1^H NMR of glycine methyl ester hydrochloride (**9**), Figure S18: ^13^C NMR of glycine methyl ester hydrochloride (**9**), Figure S19: ^1^H NMR of l-valine methyl ester hydrochloride (**10**), Figure S20: ^13^C NMR of l-valine methyl ester hydrochloride (**10**), Figure S21: ^1^H NMR of [Cu(C_10_COOH-Gly-NHC)_2_]Br (**11**), Figure S22: ^13^C NMR of [Cu(C_10_COOH-Gly-NHC)_2_]Br (**11**), Figure S23: ^1^H NMR of [Cu(C_10_COOH-Val-NHC)_2_]Br (**12**), Figure S24: ^13^C NMR of [Cu(C_10_COOH-Val-NHC)_2_]Br (**12**), Figure S25: ^1^H NMR spectrum of the polymeric catalyst (**13**), Figure S26: ^13^C NMR spectrum of the polymeric catalyst (**13**), Figure S27: ^1^H NMR of benzyl azide (**14**), Figure S28: ^13^C NMR of benzyl azide (**14**).
