# Supplementary material for: Synthesis and Mechanochemical Activity of Peptide-Based Cu(I) Bis(N-heterocyclic carbene) Complexes
Source: Biomimetics (Basel). 2019 Mar 14;4(1):24. doi: 10.3390/biomimetics4010024 (PMC6477612; doi:10.3390/biomimetics4010024)
Supplement: Supplementary file 1 [file biomimetics-04-00024-s001.pdf]

Biomimetics **2019**, *4*, 24

Supplementary Materials: Synthesis and Mechanochemical Activity of Peptide-Based Cu(I) Bis(*N*-Heterocyclic Carbene) Complexes

Sebastian Funtan, Philipp Michael and Wolfgang H. Binder *

Macromolecular Chemistry, Institute of Chemistry, Faculty of Natural Science II, Martin Luther University Halle-Wittenberg, Von-Danckelmann-Platz 4, 06120 Halle (Saale), Germany; sebastian.funtan@chemie.uni-halle.de (S.F.); philipp.michael@chemie.uni-halle.de (P.M.)

* Correspondence: wolfgang.binder@chemie.uni-halle.de; Tel.: +49-345-552-5930

Table of contents

1. Materials
2. Characterization of 11-bromoundecanoic acid methyl ester (**1**)
3. Characterization of 3-(11-methoxy-11-oxoundecyl)-1-methyl-1*H*-imidazolium bromide (**2**)
4. Characterization of [Cu(C_{10}COOMe-NHC)$_2$]Br (**3**)
5. Characterization of [Cu(C_{10}COOH-NHC)$_2$]Br (**4**)
6. Characterization of *N*-(*tert*-butoxycarbonyl)-3-bromopropylamine (**5**)
7. Characterization of 3-[3-[[(1,1-dimethylethoxy)carbonyl]amino]propyl]-1-methyl-1*H*-imidazolium bromide (**6**)
8. Characterization of [Cu(C_3NHBoc-NHC)$_2$]Br (**7**)
9. Characterization of [Cu(C_3NH_2-NHC)$_2$]Br (**8**)
10. Synthesis and characterization of glycine methyl ester hydrochloride (**9**)
11. Synthesis and characterization of L-valine methyl ester hydrochloride (**10**)
12. Characterization of [Cu(C_{10}COOH-Gly-NHC)$_2$]Br (**11**)
13. Characterization of [Cu(C_{10}COOH-Val-NHC)$_2$]Br (**12**)
14. Characterization of the polymeric catalyst (**13**)
15. Synthesis and characterization of benzyl azide (**14**)
16. Synthesis and characterization of 3-(10-carboxydecyl)-1-methyl-1*H*-imidazolium bromide

1. Materials

The following chemicals were purchased from Sigma-Aldrich: 1-methylimidazole (99%), copper(I) oxide (97%), lithium hydroxide monohydrate, 3-bromopropan-1-amine hydrobromide (98%), triethylamine (Et_3N) (99%), trifluoroacetic acid (99%), thionyl chloride 99%), *N*,*N′*-dicyclohexylcarbodiimide (DCC) (99%), pentafluorophenol (99%), *N*,*N*-diisopropylethylamine (DIPEA) (99%), and sodium azide (99.5%). 6-Bromohexanoic acid (98%) and 11-bromoundecanoic acid (99%) were purchased from Tokyo Chemical Insdustry Co., Ltd., and di-*tert*-butyl dicarbonate (99%) and *N′*-(3-dimethylaminopropyl)-*N′*-ethylcarbodiimide hydrochloride (EDC·HCl) (98%) were obtained from Carbolution Chemicals GmbH. Sodium chloride, sodium sulfate, glycine (>99%) and L-valine (>99%) were purchased from Carl Roth GmbH & Co. KG. The following chemicals were purchased from Fluka: methanesulfonic acid (98%) and benzyl bromide (98%). Hydrochloric acid (37%) and sodium bicarbonate were obtained from Grüssing GmbH, and magnesium sulfate (99%) was purchased from Alfa Aesar. High performance liquid chromatography (HPLC) grade methanol was obtained from VWR, and HPLC grade dimethylformamide (DMF) was obtained from VWR BDH Prolabo. $Ce(SO_4)\cdot 4\,H_2O$ and $(NH_4)_6Mo_7O_{24}\cdot 4\,H_2O$ were obtained from VEB. Lithium(I) bis(trifluoromethanesulfonyl)imide ($LiTf_2N$) was purchased from J&K Chemicals Ltd. Deuterated chloroform ($CDCl_3$), deuterated dimethyl sulfoxide (DMSO-d_6) and deuterated tetrahydrofuran (THF-d_8) were purchased from Chemotrade GmbH.

2. Characterization of 11-bromoundecanoic acid methyl ester (1)

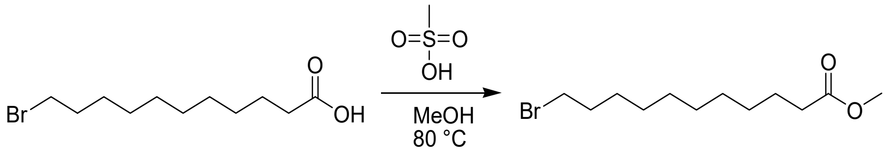

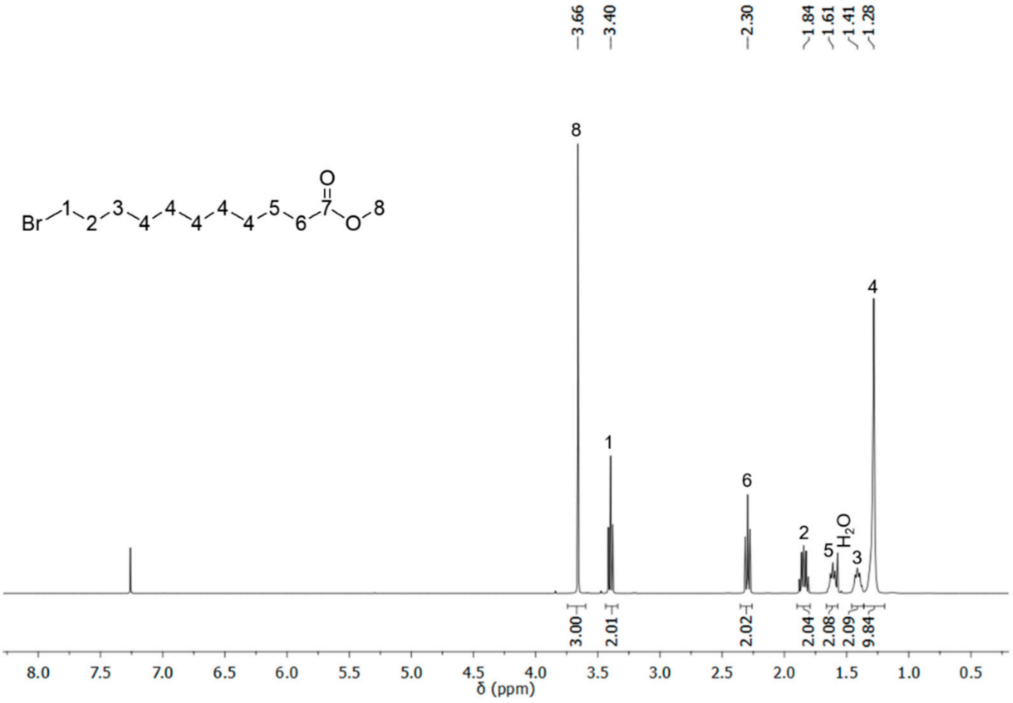

Figure S1. ^{1}H NMR spectrum of 11-bromoundecanoic acid methyl ester (**1**).

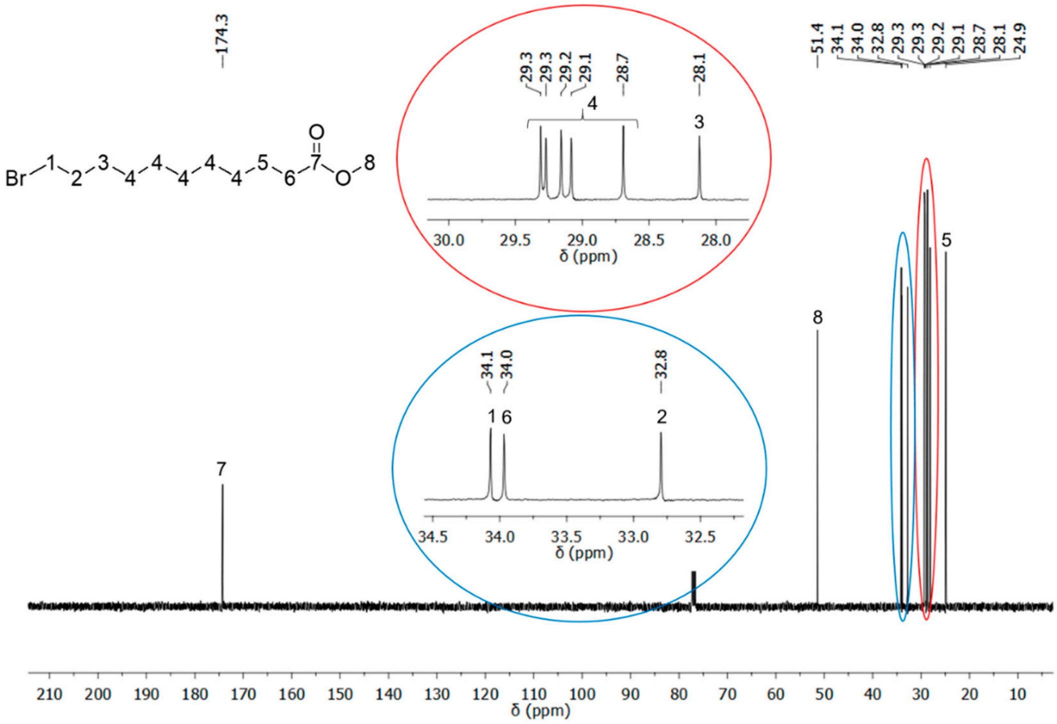

Figure S2. ^{13}C NMR spectrum of 11-bromoundecanoic acid methyl ester (**1**).

3. Characterization of 3-(11-methoxy-11-oxoundecyl)-1-methyl-1*H*-imidazolium bromide (2)

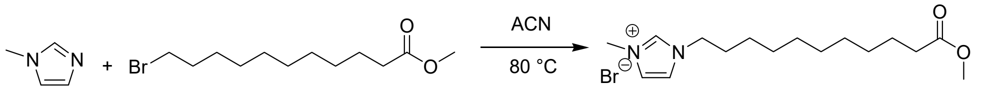

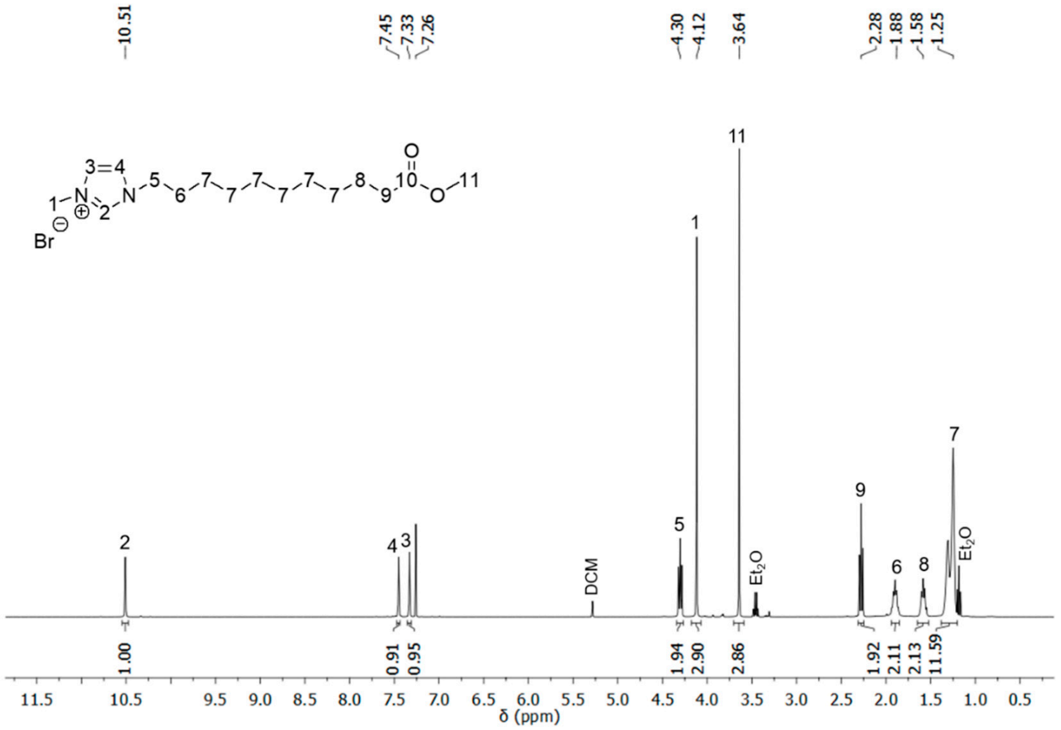

Figure S3. ^{1}H NMR spectrum of 3-(11-methoxy-11-oxoundecyl)-1-methyl-1*H*-imidazolium bromide (**2**).

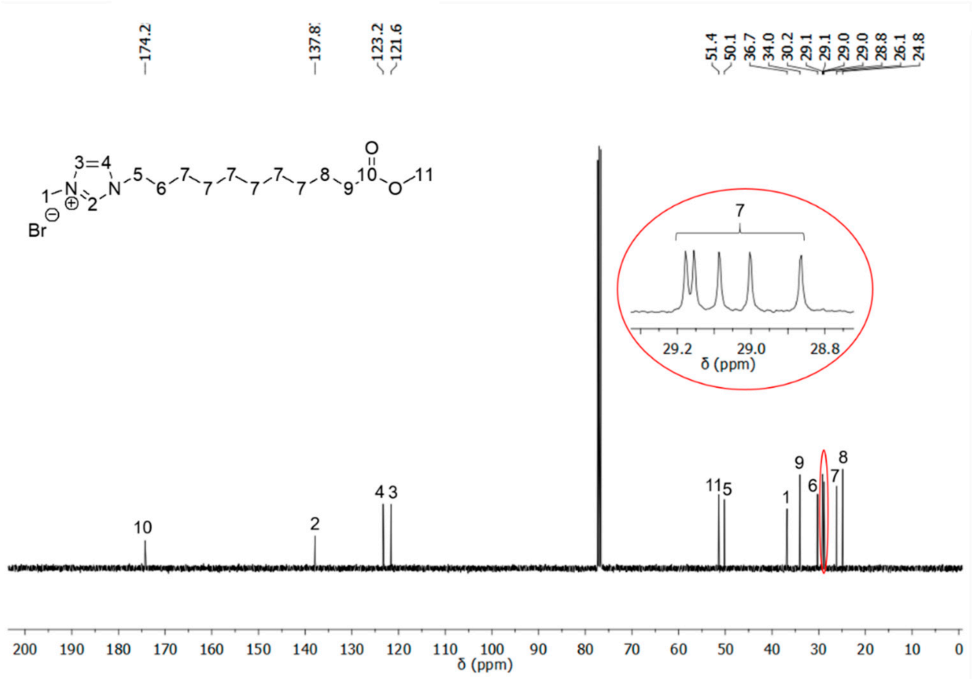

Figure S4. ^{13}C NMR spectrum of 3-(11-methoxy-11-oxoundecyl)-1-methyl-1*H*-imidazolium bromide (**2**).

4. Characterization of [Cu(C10COOMe-NHC)2]Br (3) [1]

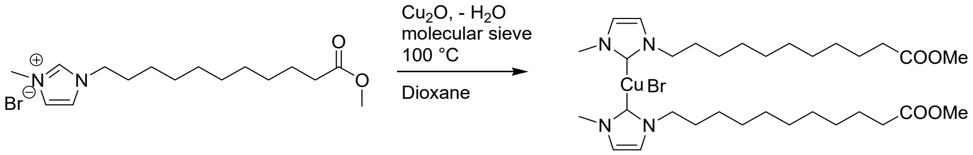

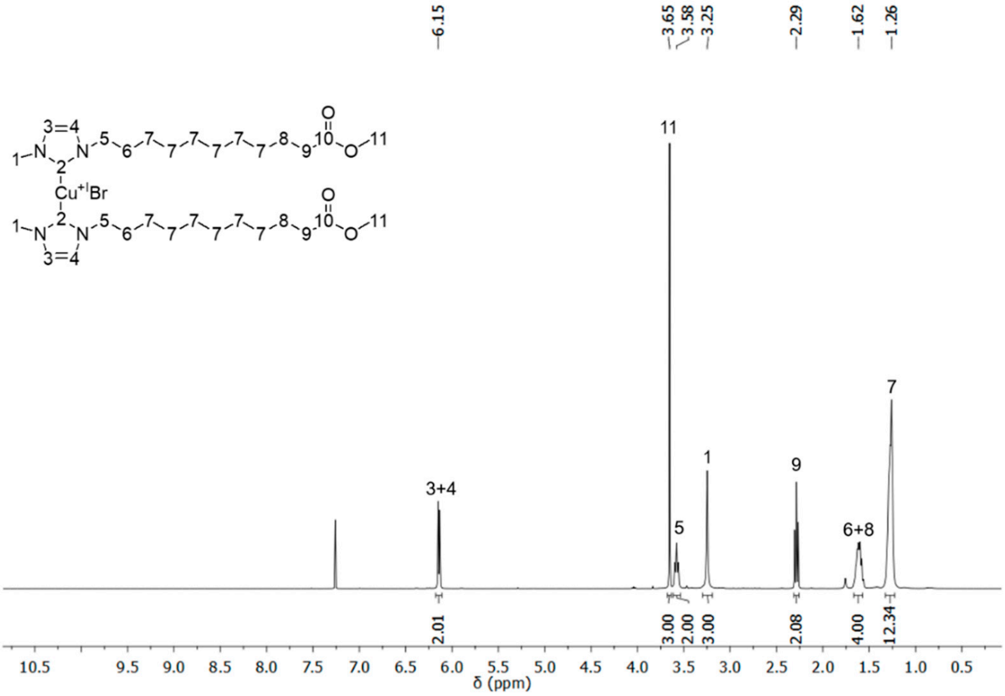

Figure S5. ^{1}H NMR spectrum of [Cu(C10COOMe-NHC)2]Br (3).

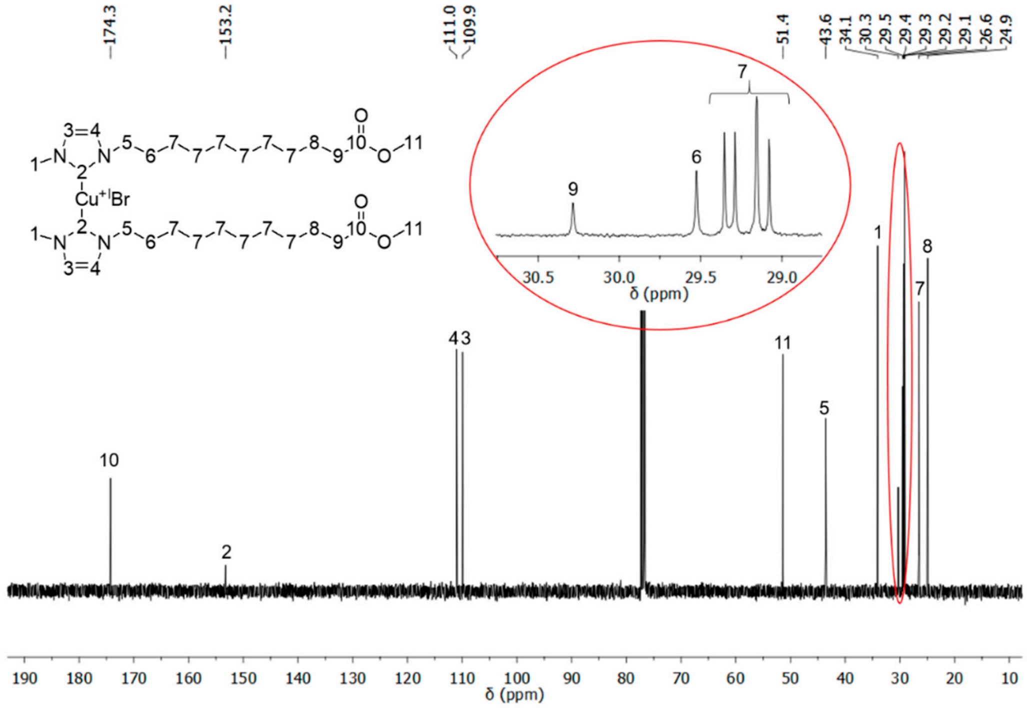

Figure S6. ^{13}C NMR spectrum of $[Cu(C_{10}COOMe\text{-}NHC)_2]Br$ (**3**).

5. Characterization of [Cu(C_{10}COOH-NHC)$_2$]Br (4) [2]

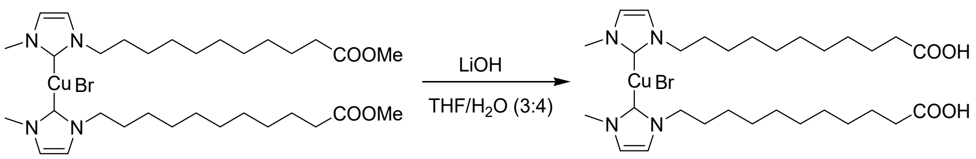

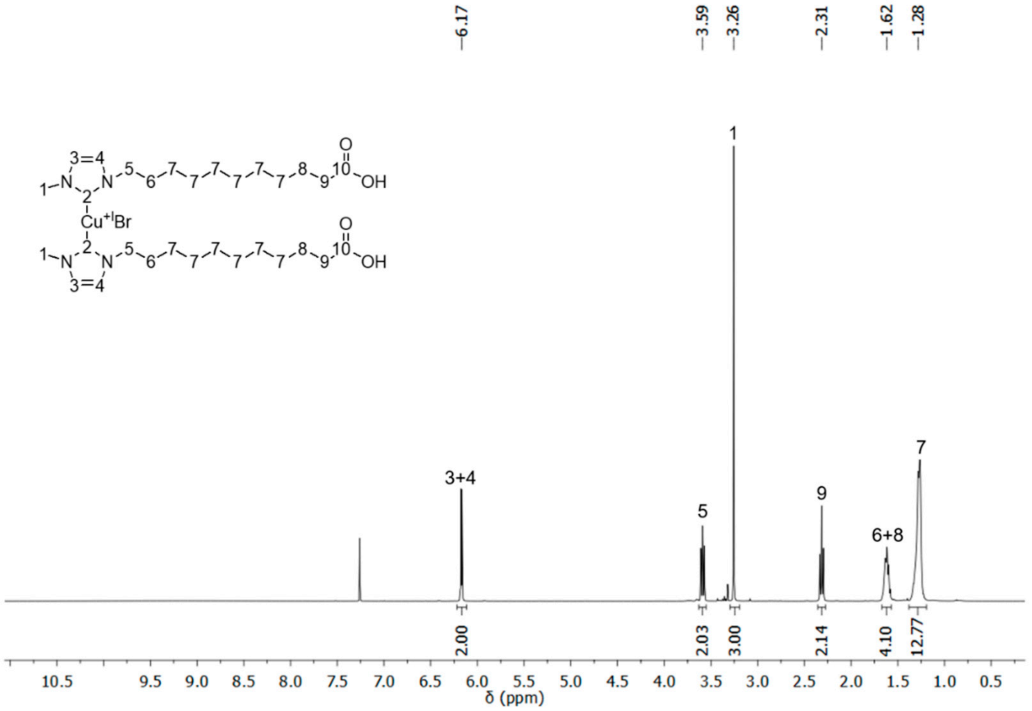

Figure S7. ^{1}H NMR spectrum of [Cu(C_{10}COOH-NHC)$_2$]Br (**4**).

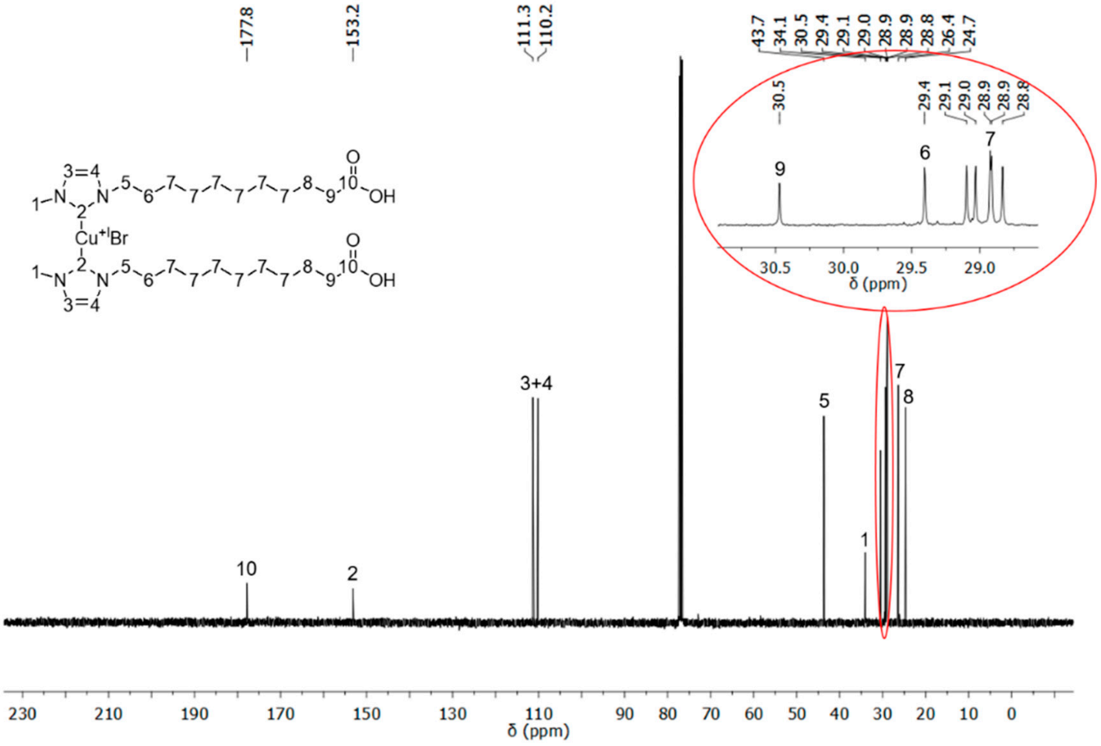

Figure S8. ^{13}C NMR spectrum of $[Cu(C_{10}COOH\text{-}NHC)_2]Br$ (**4**).

6. Characterization of *N*-(*tert*-butoxycarbonyl)-3-bromopropylamine (5) [3]

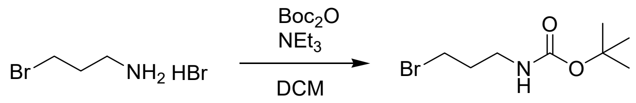

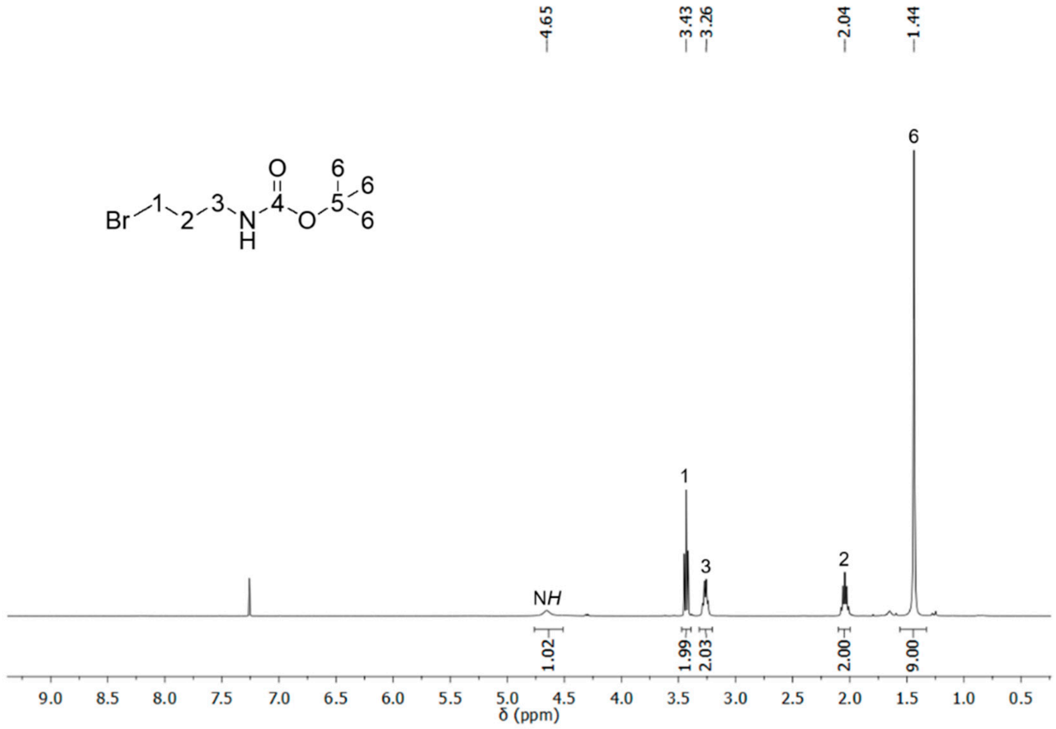

Figure S9. ^{1}H NMR spectrum of *N*-(*tert*-butoxycarbonyl)-3-bromopropylamine (**5**).

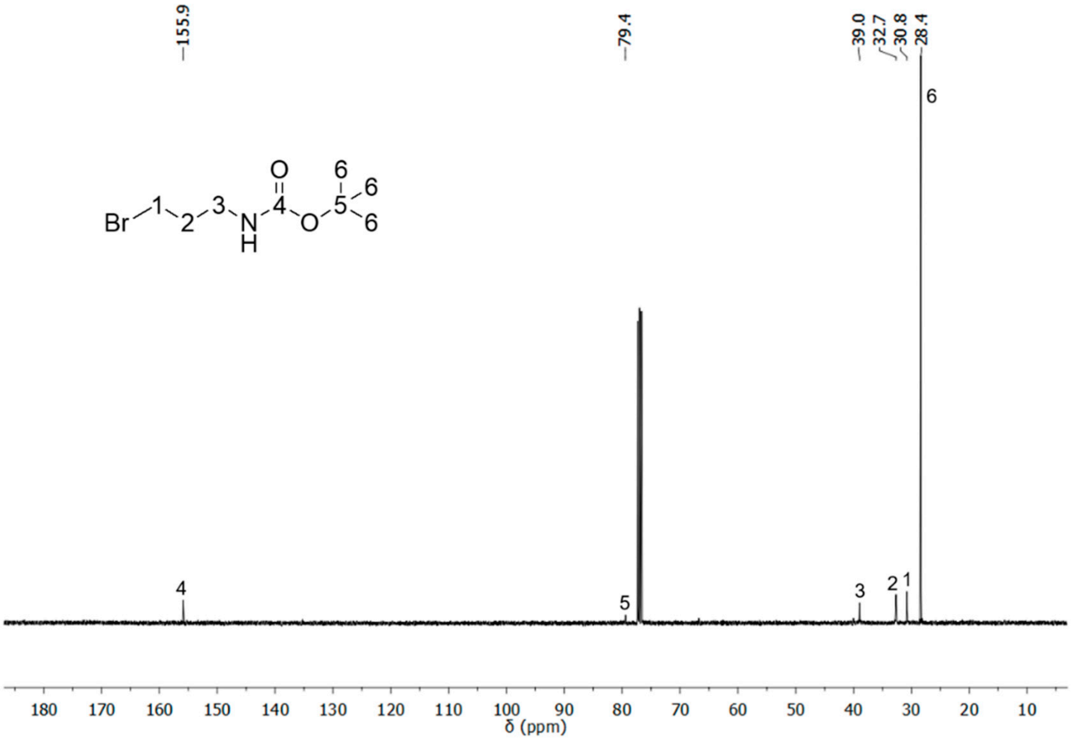

Figure S10. ^{13}C NMR spectrum of *N*-(*tert*-butoxycarbonyl)-3-bromopropylamine (**5**).

7. Characterization of 3-[3-[[(1,1-dimethylethoxy)carbonyl]amino]propyl]-1-methyl-1*H*-imidazolium bromide (6)

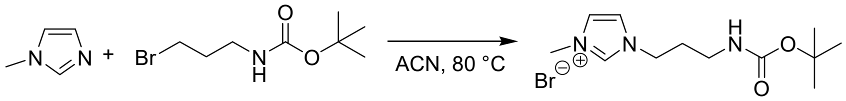

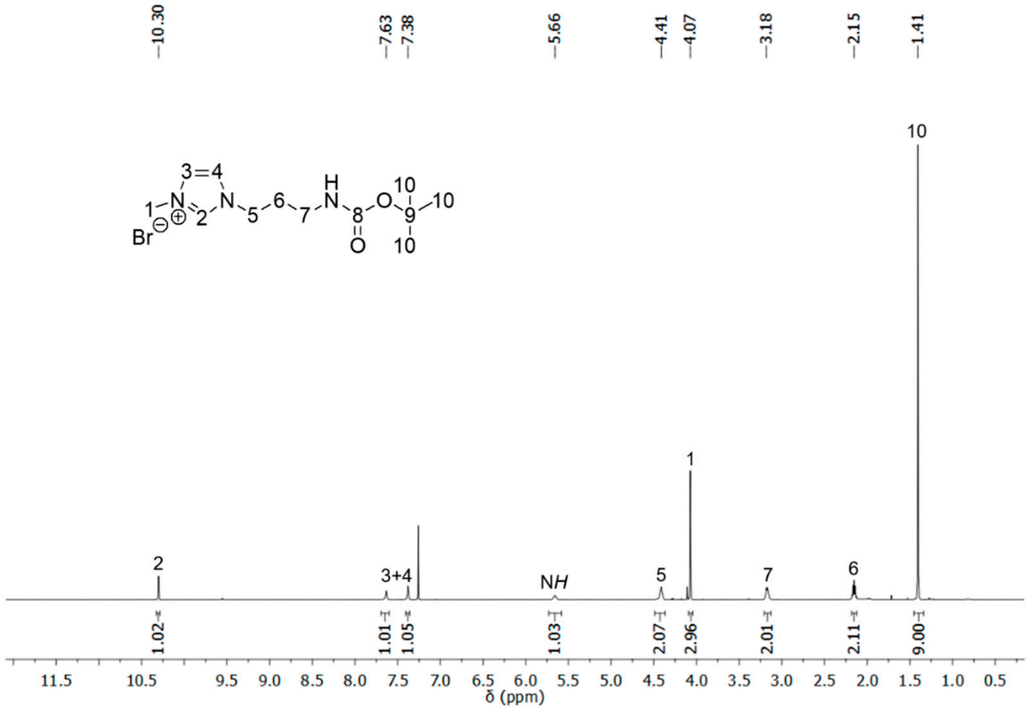

Figure S11. ^{1}H NMR spectrum of 3-[3-[[(1,1-dimethylethoxy)carbonyl]amino]propyl]-1-methyl-1*H*-imidazolium bromide (**6**).

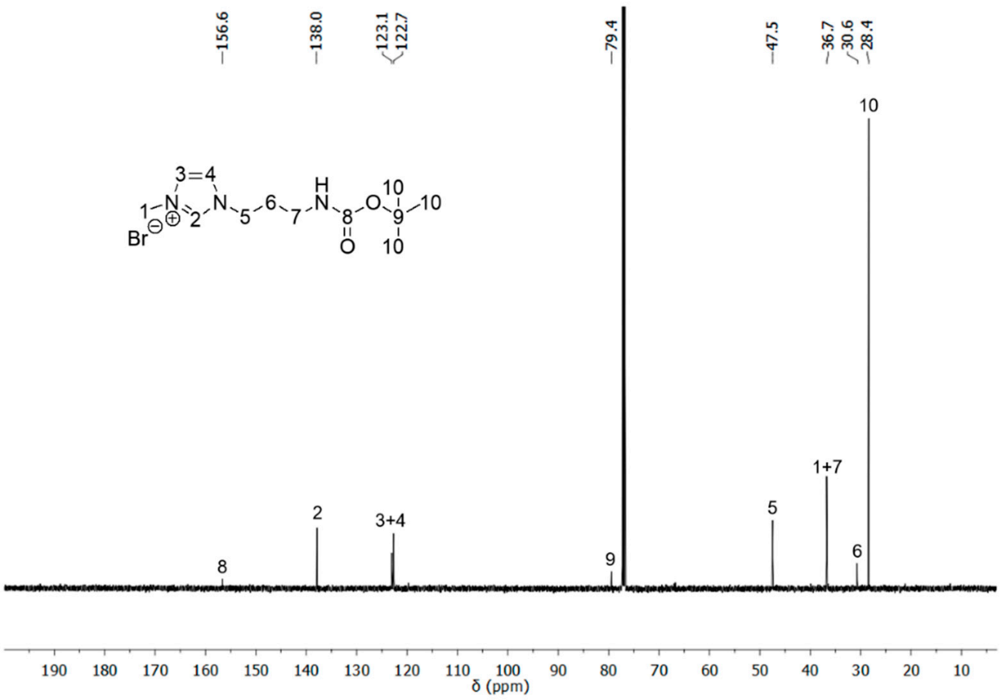

Figure S12. ^{13}C NMR spectrum of 3-[3-[[(1,1-dimethylethoxy)carbonyl]amino]propyl]-1-methyl-1*H*-imidazolium bromide (**6**).

8. Characterization of [Cu(C_3NHBoc-NHC)$_2$]Br (7) [1]

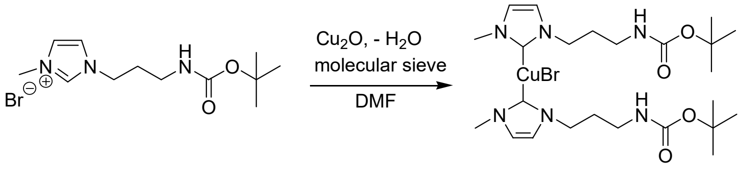

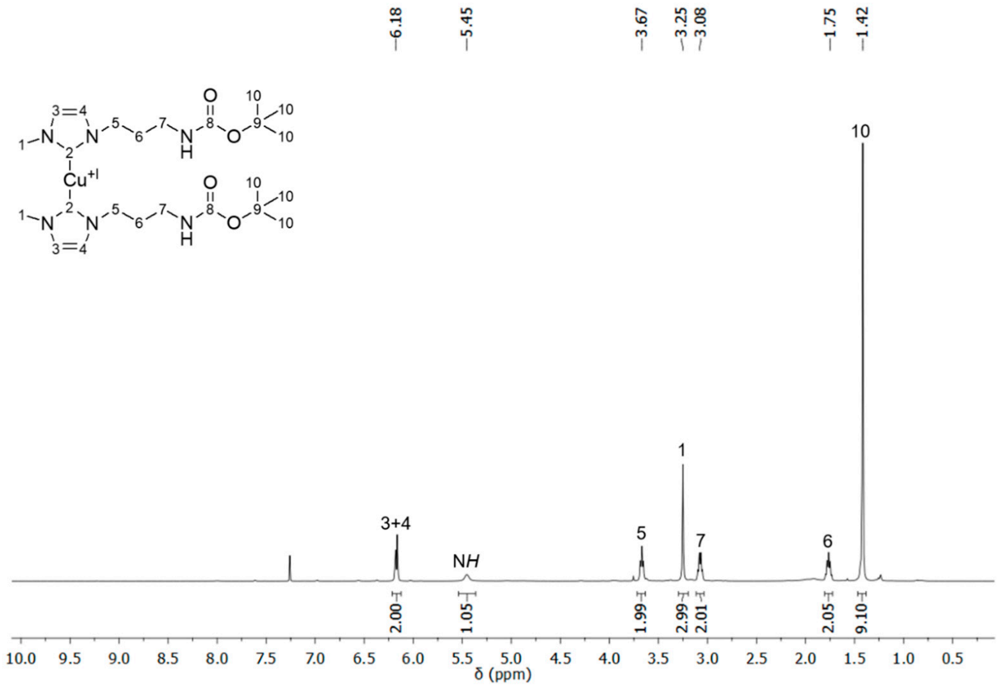

Figure S13. ^{1}H NMR spectrum of [Cu(C_3NHBoc-NHC)$_2$]Br (**7**).

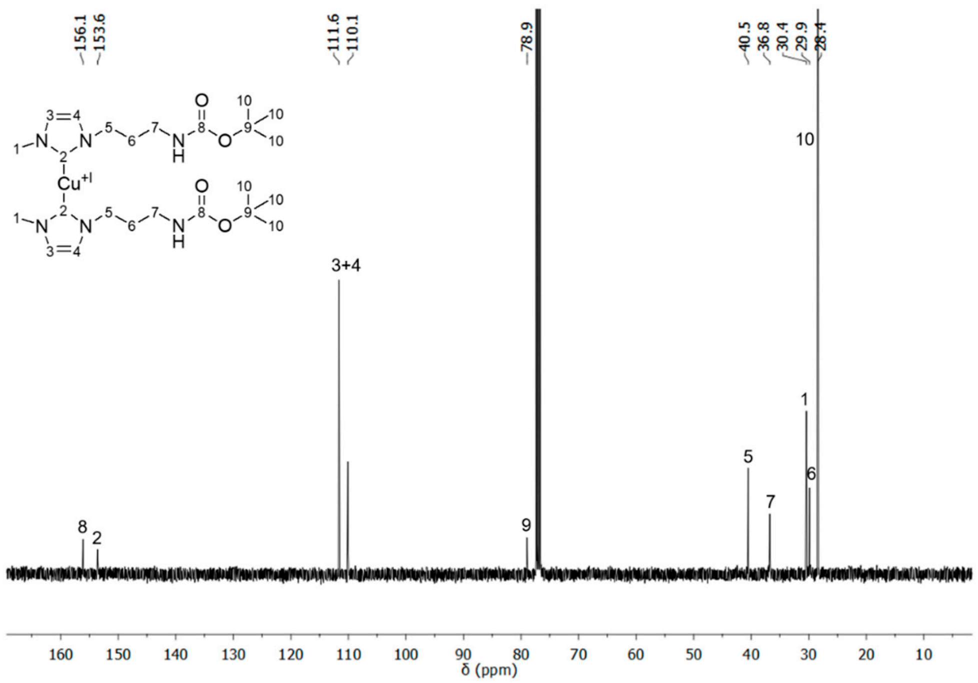

Figure S14. ^{13}C NMR spectrum of $[Cu(C_3NHBoc\text{-}NHC)_2]Br$ (**7**).

9. Characterization of [Cu(C3NH2-NHC)2]Br (8)

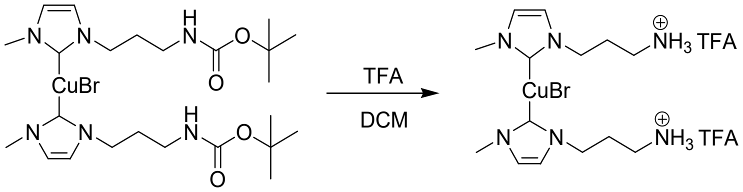

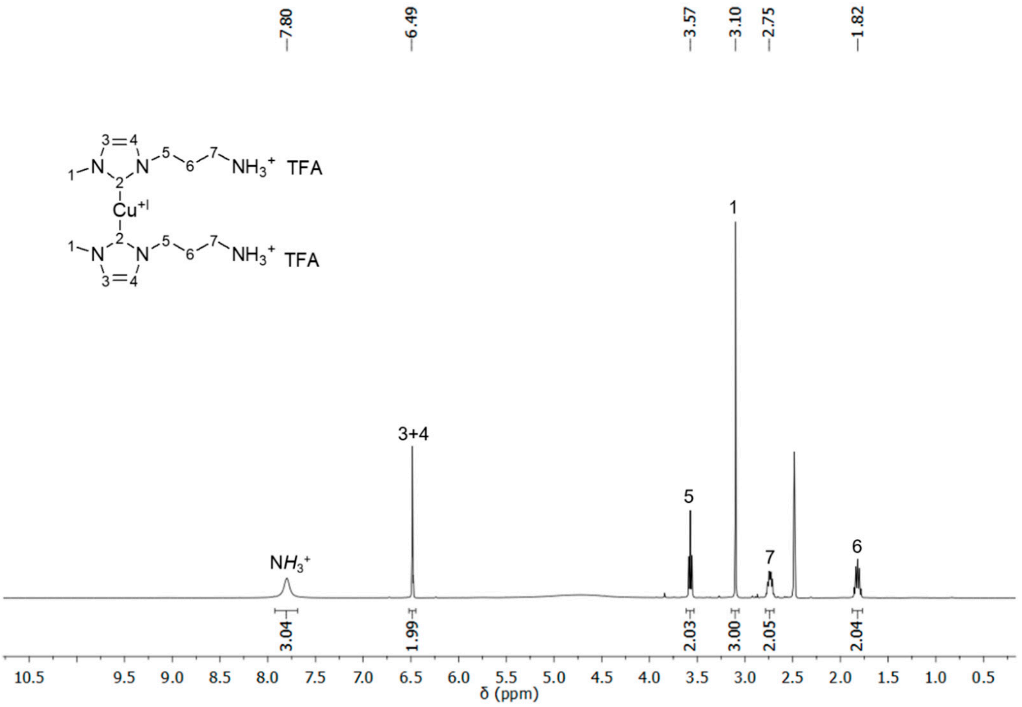

Figure S15. ^{1}H NMR spectrum of [Cu(C3NH2-NHC)2]Br (**8**).

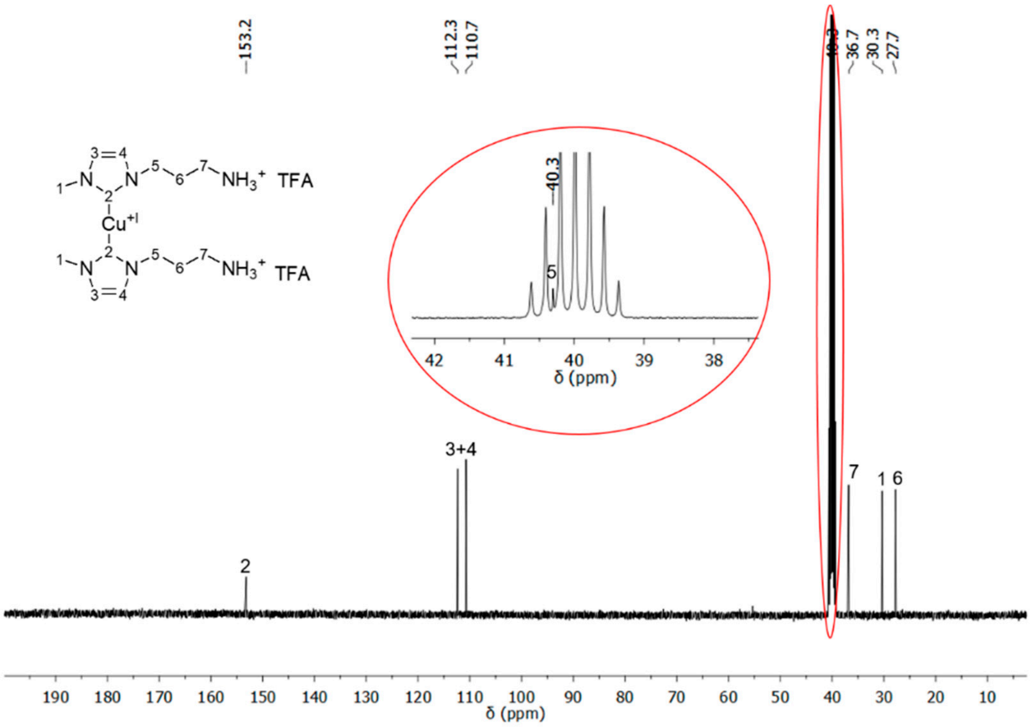

Figure S16. ^{13}C NMR spectrum of [Cu(C$_3$NH$_2$-NHC)$_2$]Br (**8**).

10. Synthesis and characterization of glycine methyl ester hydrochloride (9) [4]

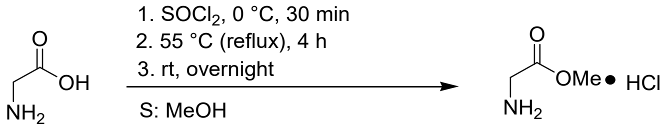

Glycine (5.0 g, 66.6 mmol) was dissolved in MeOH (65.0 mL) and cooled down to 0 °C before $SOCl_2$ (8.70 g, 5.3 mL, 73.3 mmol) was slowly added. Subsequently the reaction mixture was heated to 55 °C for 4 h and then was cooled down to room temperature (rt). After stirring overnight, the solvent and the excess of $SOCl_2$ were removed under vacuum. Recrystallization from MeOH yielded the pure product as white crystalline needles. Yield: 6.58 g, 52.37 mmol, 79%.

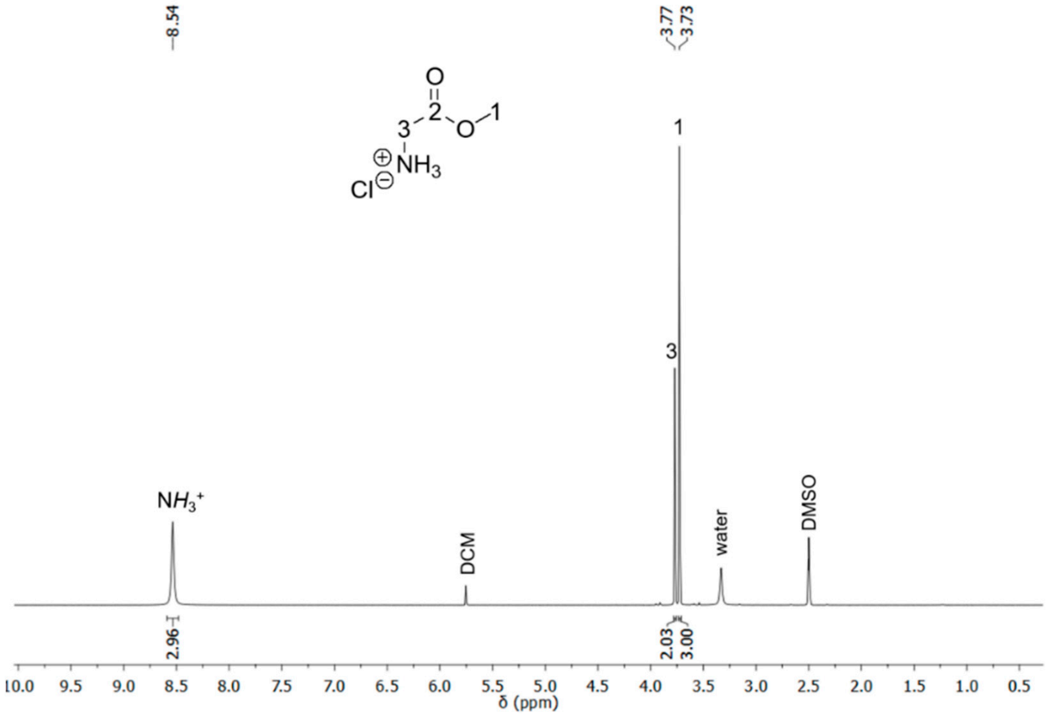

Figure S17. ^{1}H NMR spectrum of glycine methyl ester hydrochloride (**9**).

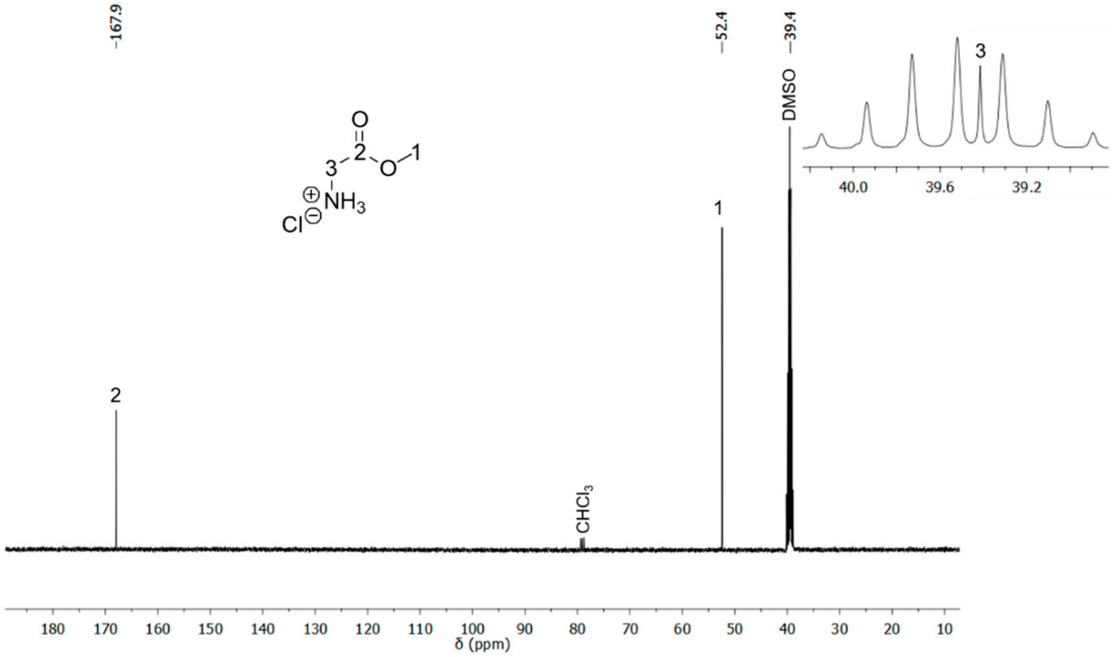

Figure S18. ^{13}C NMR spectrum of glycine methyl ester hydrochloride (**9**).

11. Synthesis and characterization of L-valine methyl ester hydrochloride (10) [5]

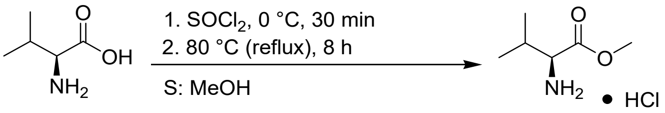

L-Valine (5.0 g, 42.67mmol) was dissolved in dry methanol (60.0 mL) and was cooled down to 0 °C. Freshly distilled thionyl chloride (7.6 g, 4.64 mL, 64.2 mmol) was slowly added and after stirring for 30 min the temperature was increased to 80 °C and the mixture was refluxed for another 8 h. After removal of the solvent and the excess of thionyl chloride, the product was recrystallized from methanol followed by filtration to obtain the pure product as a colorless powder. Yield: 6.21 g, 37.1 mmol, 87%.

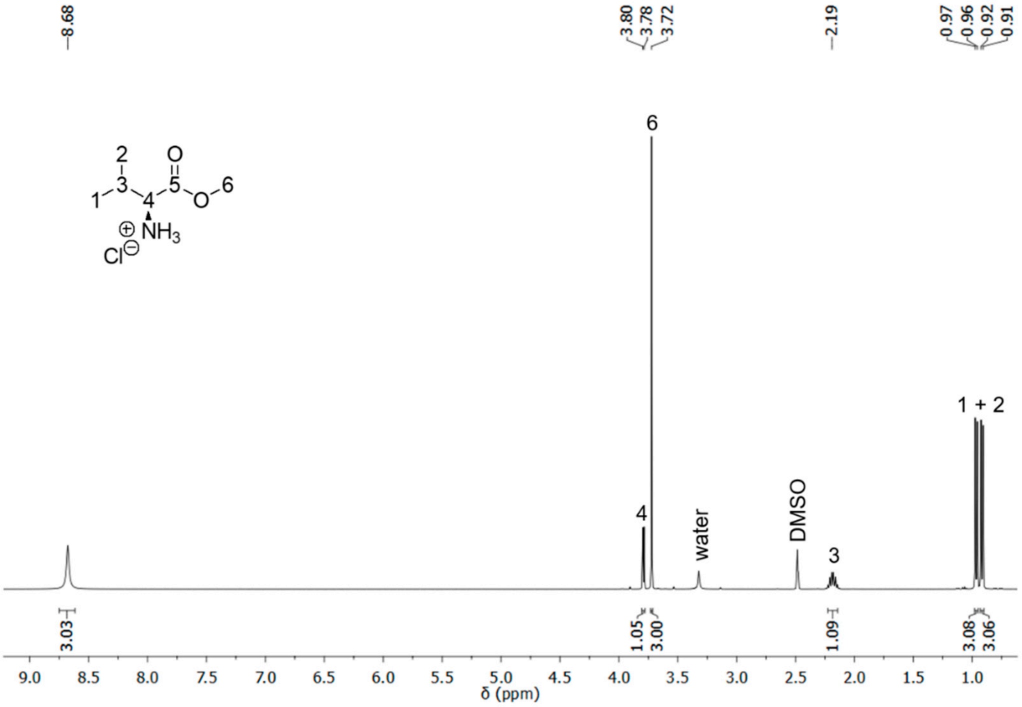

Figure S19. ^{1}H NMR spectrum of L-valine methyl ester hydrochloride (**10**).

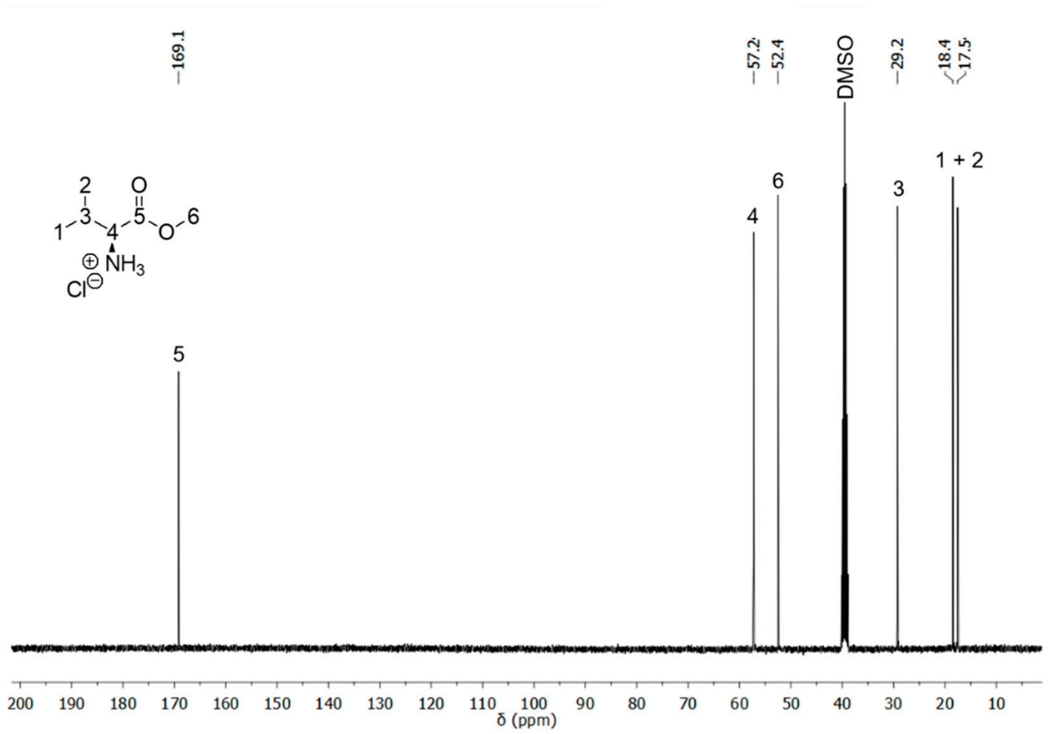

Figure S20. ^{13}C NMR spectrum of L-valine methyl ester hydrochloride (**10**).

12. Characterization of [Cu(C10COOH-Gly-NHC)2]Br (11) [6,7]

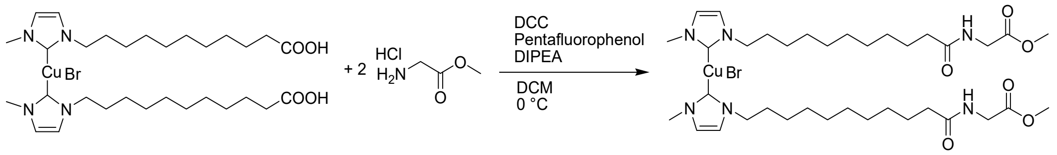

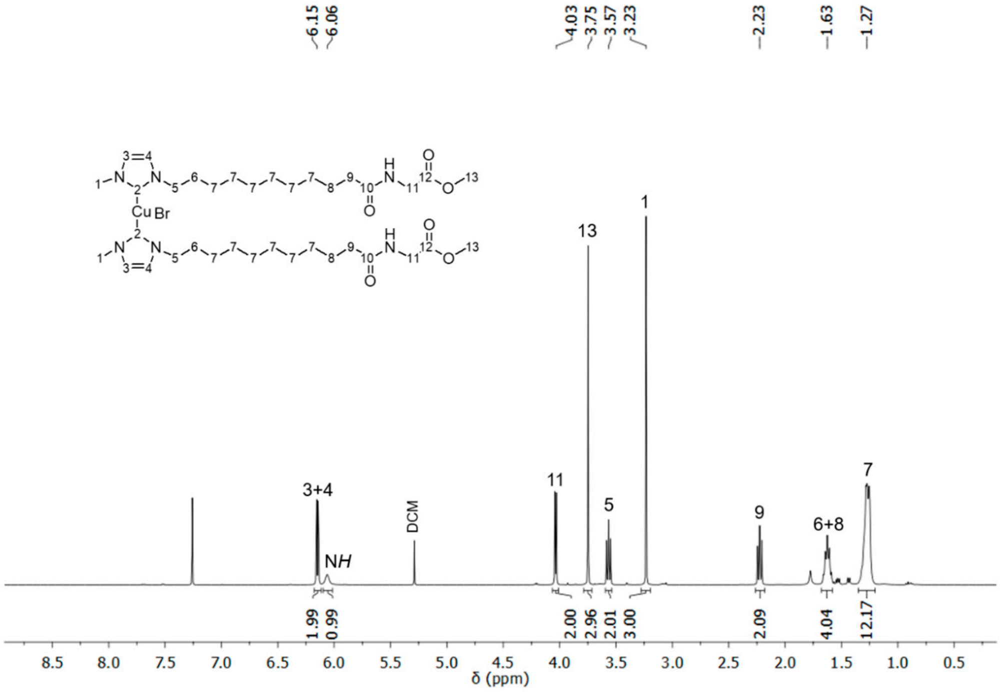

Figure S1. ^{1}H NMR spectrum of [Cu(C10COOH-Gly-NHC)2]Br (**11**).

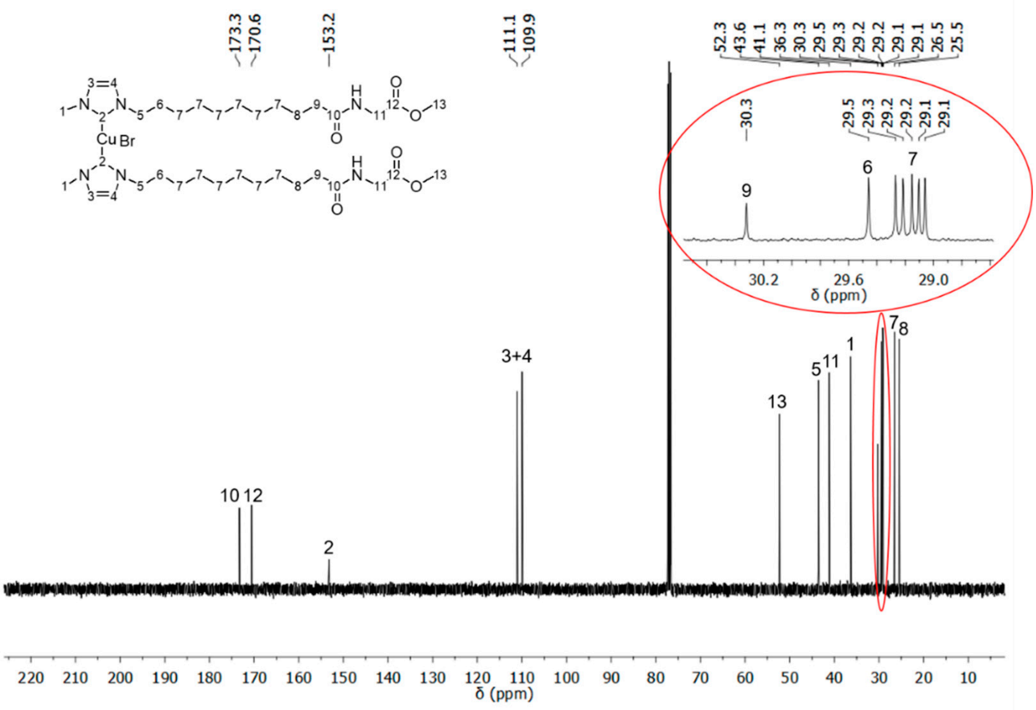

Figure S22. ^{13}C NMR spectrum of $[Cu(C_{10}COOH\text{-}Gly\text{-}NHC)_2]Br$ (**11**).

13. Characterization of [Cu(C10COOH-Val-NHC)2]Br (12) [6,7]

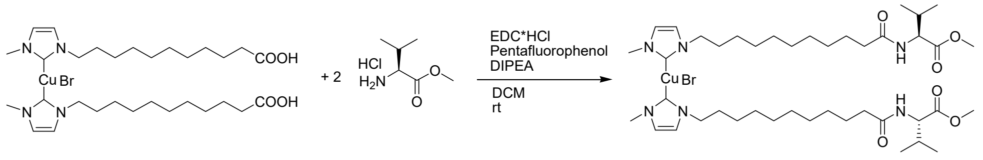

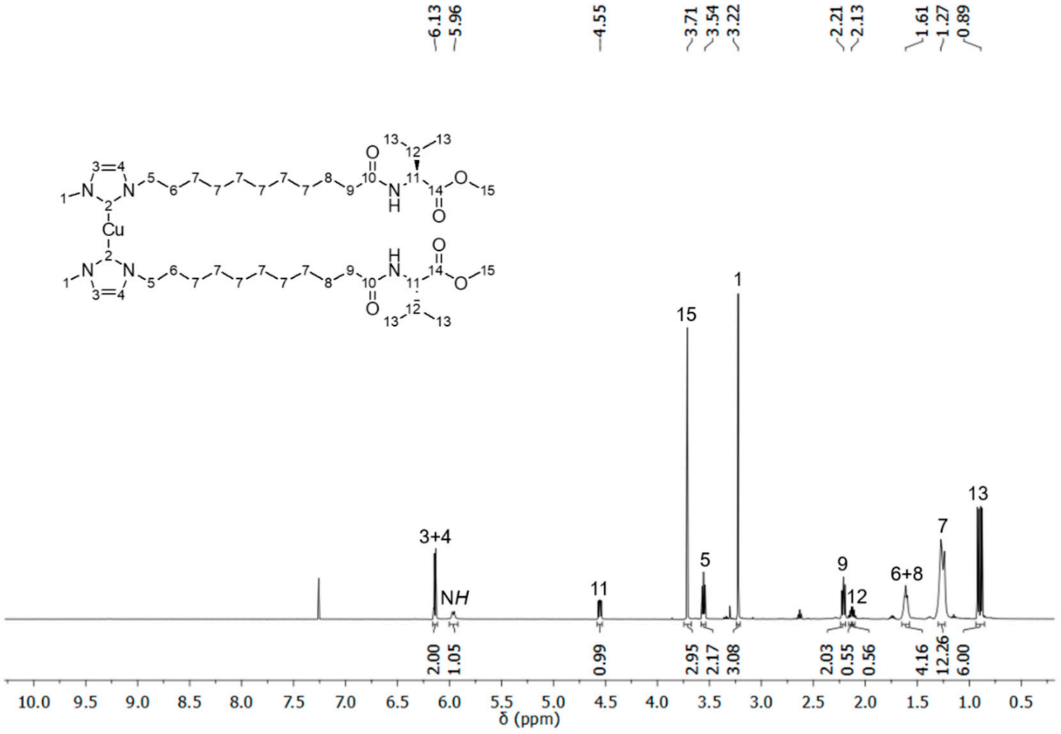

Figure S23. ^{1}H NMR spectrum of [Cu(C10COOH-Val-NHC)2]Br (**12**).

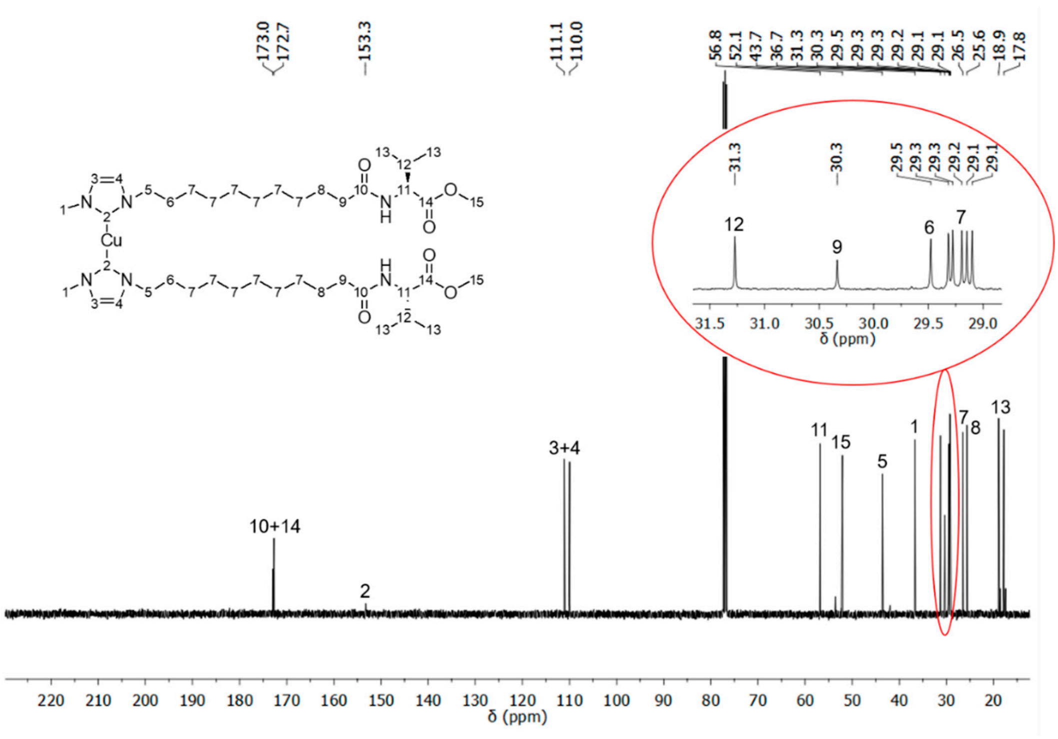

Figure S24. ^{13}C NMR spectrum of [Cu(C$_{10}$COOH-Val-NHC)$_2$]Br (**12**).

14. Characterization of the polymeric catalyst (13) [6,7]

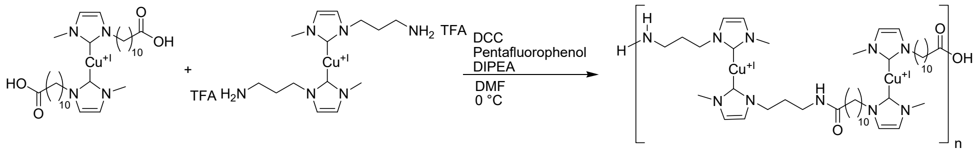

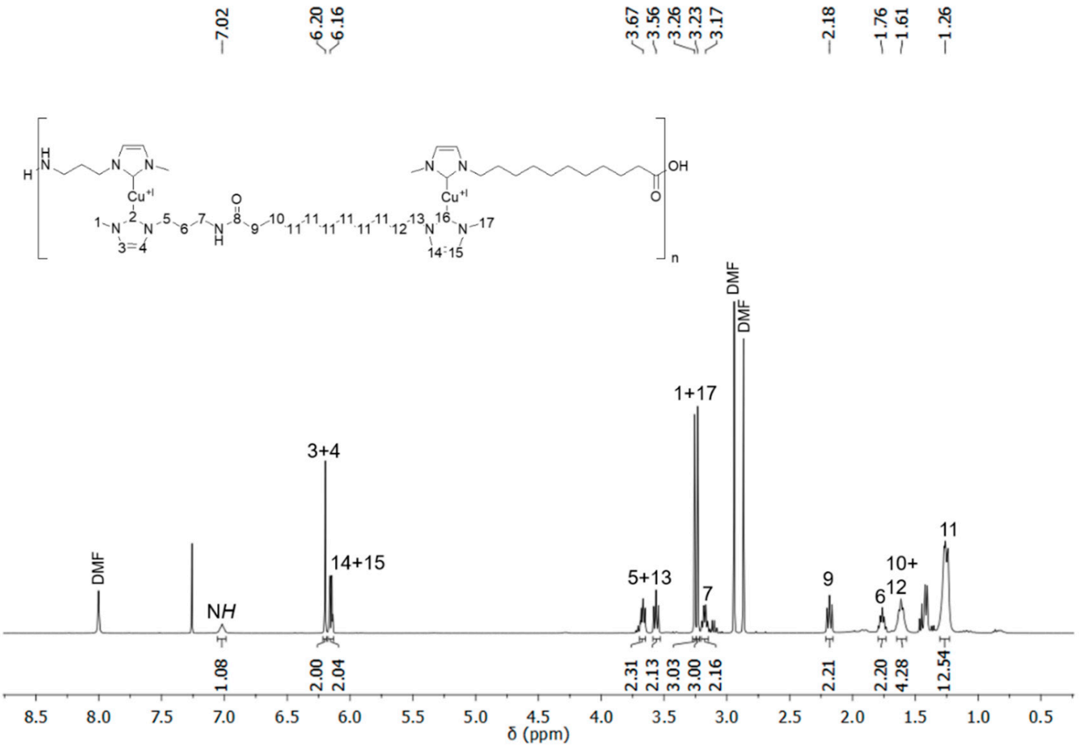

Figure S25. ^{1}H NMR spectrum of the polymeric catalyst **13**.

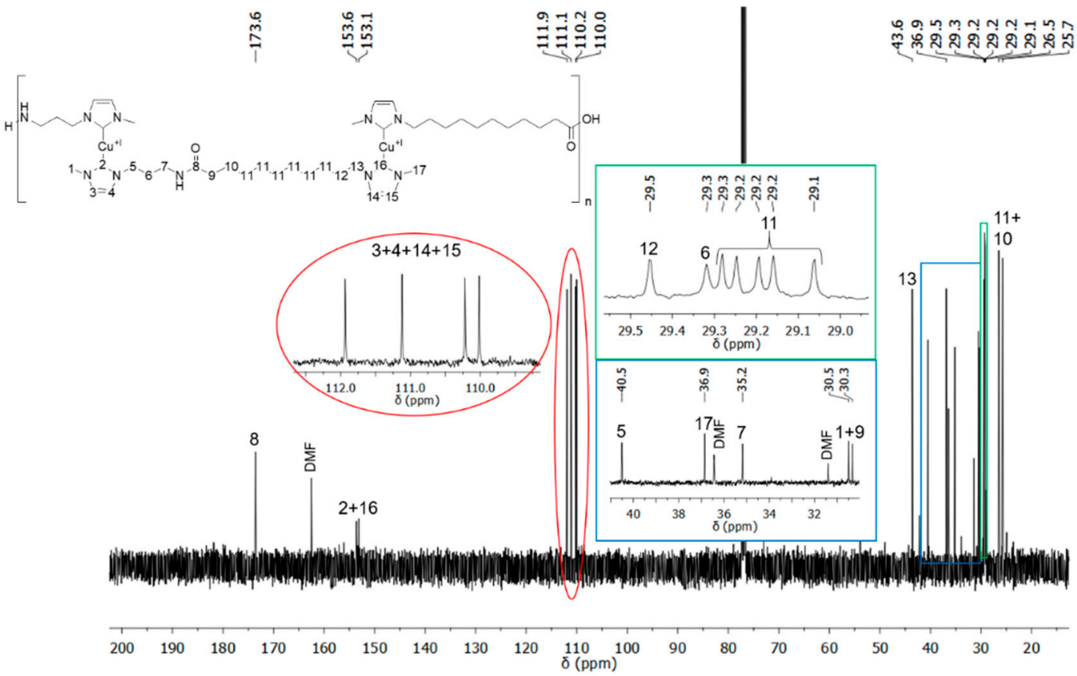

Figure S26. ^{13}C NMR spectrum of the polymeric catalyst **13**.

15. Synthesis and characterization of benzyl azide (14) [8]

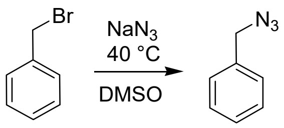

Sodium azide (5.5 g, 84.7 mmol) and benzyl bromide (**14**) (13.2 g, 9.17 mL, 77.0 mmol) were dissolved in DMSO (170.0 mL). The reaction mixture was stirred at 40 °C for 90 h. Afterward, water (50.0 mL) was added and the reaction was further stirred, until it was cooled down to room temperature. The mixture was extracted with Et_2O (3 × 30.0 mL) and the combined organic phases were washed with water (2 ×50.0 mL) and brine (50.0 mL). Subsequently the organic phase was dried over Na_2SO_4, filtered and the solvent was removed under reduced pressure. Yield: 9.45 g, 71.0 mmol, 92%.

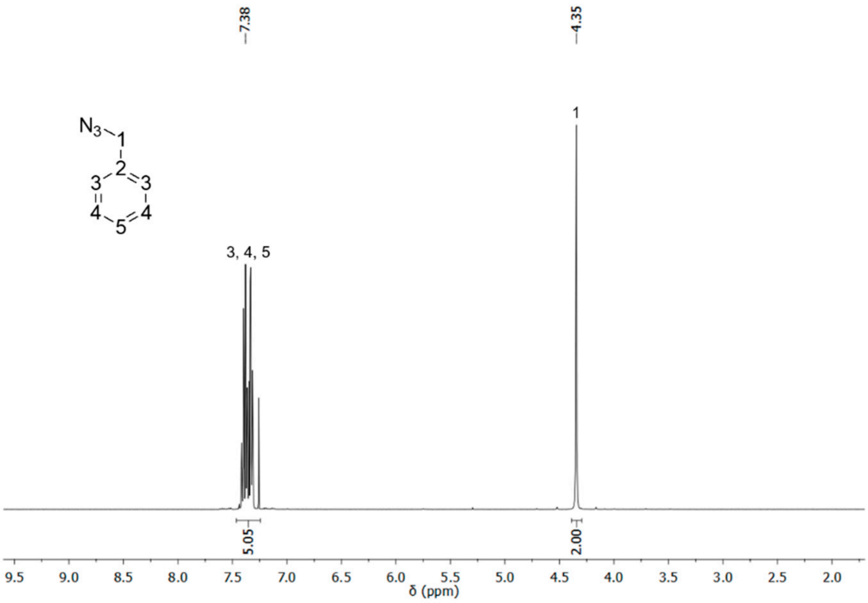

Figure S27. ^{1}H NMR spectrum of benzyl azide (**14**).

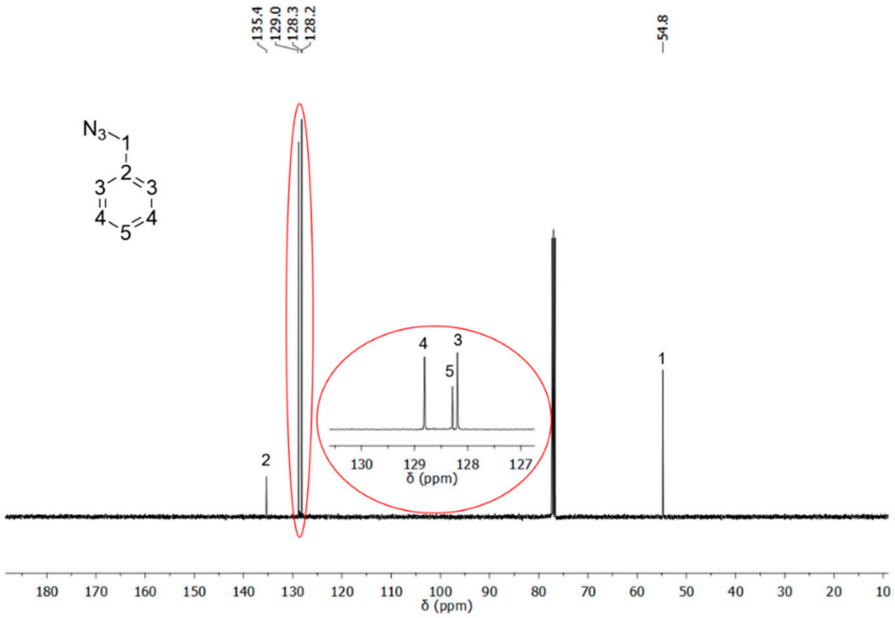

Figure S28. ^{13}C NMR spectrum of benzyl azide (**14**).

16. Synthesis and characterization of 3-(10-carboxydecyl)-1-methyl-1*H*-imidazolium bromide

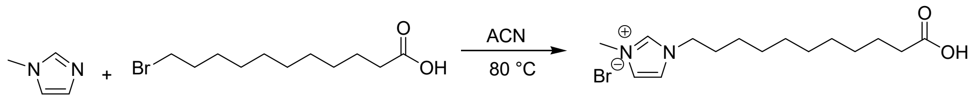

11-Bromoundecanoic (10.0 g, 37.73 mmol) and 1-methylimidazole (3.27 g, 3.13 mL, 39.62 mmol) were dissolved in ACN (27.0 mL). The reaction mixture was stirred for 48 h at 80 °C. Afterward, the solvent was removed and the crude product was washed with Et_2O (2 × 20.0 mL) and cold ACN (4 × 10.0 mL). The pure product was obtained as a white, crystalline solid. Yield: 11.63 g, 33.49 mmol, 89%.

^1H NMR (400 MHz, DMSO-d_6, 27 °C): δ (ppm) = 11.94 (s, 1H), 9.13 (s, 1H), 7.76 (s, 1H), 7.69 (s, 1H), 4.14 (t, 2H), 3.83 (s, 3H), 2.16 (t, 2H), 1.75 (m, 2H), 1.46 (m, 2H), 1.22 (m, 12H); ^{13}C NMR (100 MHz, DMSO-d_6, 27 °C): δ (ppm) = 174.9, 136.9, 124.0, 122.7, 49.2, 36.2, 34.1, 29.8, 29.2, 29.2, 29.1, 29.0, 28.8 , 25.9, 25.0.

References

1. Chun, J.; Lee, H.S.; Jung, I.G.; Lee, S.W.; Kim, H.J.; Son, S.U. Cu_2O: A versatile reagent for base-free direct synthesis of NHC-copper complexes and decoration of 3D-MOF with coordinatively unsaturated NHC-copper species. *Organometallics* **2010**, *29*, 1518–1521, doi:10.1021/om900768w.
2. Chen, Y.; Guan, Z. Bioinspired modular synthesis of elastin-mimic polymers to probe the mechanism of elastin elasticity. *J. Am. Chem. Soc.* **2010**, *132*, 4577–4579, doi:10.1021/ja9104446.
3. Roy, S.; Eastman, A.; Gribble, G.W. Synthesis of bisindolylmaleimides related to GF109203x and their efficient conversion to the bioactive indolocarbazoles. *Org. Biomol. Chem.* **2006**, *4*, 3228–3234, doi:10.1039/B607504E.
4. Li, F.; Bravo-Rodriguez, K.; Phillips, C.; Seidel, R.W.; Wieberneit, F.; Stoll, R.; Doltsinis, N.L.; Sanchez-Garcia, E.; Sander, W. Conformation and dynamics of a cyclic disulfide-bridged peptide: Effects of temperature and solvent. *J. Phys. Chem. B* **2013**, *117*, 3560–3570, doi:10.1021/jp4007334.
5. Tauchman, J.; Císařová, I.; Štěpnička, P. Chiral phosphanylferrocenecarboxamides with amino acid pendant groups as ligands for Cu-mediated asymmetric conjugate additions of diethylzinc to chalcones—Structural characterisation of precursors to the Cu catalyst. *Eur. J. Org. Chem.* **2010**, *2010*, 4276–4287, doi:10.1002/ejoc.201000480.
6. Meneses, C.; Nicoll, S.L.; Trembleau, L. Multigram-scale synthesis of short peptides via a simplified repetitive solution-phase procedure. *J. Org. Chem.* **2010**, *75*, 564–569, doi:10.1021/jo902116p.
7. Cinzia, C.; Anna, B. Synthesis of α-*N*-linked glycopeptides. *Eur. J. Org. Chem.* **2011**, *2011*, 3911–3919, doi:10.1002/ejoc.201100124.
8. Alvarez, S.G.; Alvarez, M.T. A practical procedure for the synthesis of alkyl azides at ambient temperature in dimethyl sulfoxide in high purity and yield. *Synthesis* **1997**, *1997*, 413–414, doi:10.1055/s-1997-1206.
